# Ampyrone is a direct agonist of human tyrosinase and a potential therapeutic for hypopigmentation disorders

**DOI:** 10.1172/jci.insight.202947

**Published:** 2026-06-18

**Authors:** Monika B. Dolinska, Yuhong Wang, Nathan P. Coussens, Vijay K. Kalaskar, Zuhal Eraslan, Samuel J. Grondin, Joseph Bonica, Sarah Toay, Matthew D. Hall, Min Shen, Matthew Boxer, Qiuying Chen, Steven S. Gross, Nabeel Attarwala, Yingyos Jittayasothorn, Ramakrishna P. Alur, Dhyanam Shukla, Robin Kee, Charles DeYoung, Cuilee Sha, David R. Adams, Stacie K. Loftus, Tiziana Cogliati, Yuri V. Sergeev, Jonathan H. Zippin, Brian P. Brooks

**Affiliations:** 1 National Eye Institute, National Institutes of Health, Bethesda, Maryland.; 2 National Center for Advancing Translational Sciences, National Institutes of Health, Rockville, Maryland, USA.; 3 Department of Dermatology and; 4 Department of Pharmacology, Weill Cornell School of Medicine, New York, New York, USA.; 5 National Human Genome Research Institute, National Institutes of Health, Bethesda, Maryland.; 6 Office of the Clinical Director, National Human Genome Research Institute, Bethesda, Maryland, USA.; 7 Englander Institute for Precision Medicine, Weill Cornell School of Medicine, New York, New York, USA.

**Keywords:** Dermatology, Ophthalmology, Drug screens, Drug therapy

## Abstract

Significant loss of pigmentation can increase visual disability, skin cancer risk, and psychosocial stress. Tyrosinase (TYR) catalyzes the first and rate-limiting step of melanin synthesis. Inhibitors of TYR are well established and are currently used in clinical settings; however, there is a dearth of direct activators of TYR. Here, using a human TYR construct, we developed high-throughput screening methods, in cell confirmatory assays employing ^13^C-tyrosine tracing, and computational analysis techniques, and identified ampyrone (4-aminoantipyrine) as a TYR activator. Ampyrone increased the in vitro catalytic activity of the human recombinant intramelanosomal domain of TYR (hTYR) and its hypomorphic variant, Pro406Leu (P406L), a cause of oculocutaneous albinism type 1B (OCA1B). Moreover, ampyrone induced melanin synthesis in both WT and OCA1B human melanocytes, mouse OCA2 melanocytes, as well as 3-dimensional (3D) human skin cultures. Computational studies provided additional insight into the effects of direct TYR agonists on enzyme activity. Our results identify ampyrone as a lead candidate for TYR activation, potentially supporting the development of therapies for patients with genetic and acquired diseases of hypopigmentation.

## Abstract 2

A drug-like compound called ampyrone increases melanin production by activating tyrosinase, showing promise as a future treatment for reduced pigmentation disorders.

## Introduction

Pigmentation of the hair, eyes, and skin is not only a notable phenotypic characteristic, but it also protects from ultraviolet (UV) radiation and affects visual acuity. Disorders of pigmentation can be acquired (e.g., postinflammatory, metabolic abnormalities) or genetic (e.g., oculocutaneous albinism [OCA]) (1–3). In many acquired instances, these pigmentary disorders are transient in nature, but in some individuals, the effects can last for many years or never resolve. Cutaneous hypopigmentation increases skin cancer risk and can lead to negative psychosocial effects, including social stigmatization, reduced self-esteem, and heightened self-consciousness (4, 5). Individuals with OCA may present with an uncorrectable visual disability. Despite the morbidity associated with hypopigmentation diseases of the skin and eyes, there are limited therapeutic approaches.

Pigmentation in animals is primarily a reflection of melanin concentration. Melanin is a polymer synthesized within specialized organelles called melanosomes. In humans, melanosomes are most prominently present in 2 cell types: (a) the neural crest–derived melanocytes present in the hair, epidermis, meninges of the brain, stria vascularis of the ear, and the choroid and iris of the eye as well as (b) the neuroectoderm-derived pigmented epithelial cells, such as retinal pigment epithelium (RPE) cells of the eye (6). Although these cell types represent the primary sites of melanin synthesis, melanin can also be detected in other tissues or cell types under specific developmental, physiological, or pathological conditions. Tyrosinase (TYR) is a type 1 transmembrane, copper-containing glycoenzyme that catalyzes the initial and rate-limiting steps of melanin production in melanosomes including the direct oxidation of L-tyrosine to dopaquinone (monophenolase activity) and the oxidation of L-DOPA to dopaquinone (diphenol oxidase activity) (7). Over the last few decades, numerous cosmeceutical and pharmaceutical companies have used nonvertebrate TYR to screen for small molecules that alter melanin synthesis. This approach has led to the discovery of various TYR inhibitors and effective treatments for hyperpigmentation disorders of the skin, such as postinflammatory hyperpigmentation and melasma (8, 9). In contrast, activators of melanin synthesis have remained comparatively elusive (10). Although several compounds have been reported to stimulate melanin synthesis, most act indirectly by upregulating TYR expression or modulating upstream signaling pathways rather than directly enhancing catalytic activity of the enzyme (11–13). Direct activators of TYR would therefore have significant clinical utility in multiple forms of albinism and could represent a therapeutic approach for improving pigmentation in those with fair skin and increased risk of skin cancer. In addition, TYR agonists would be helpful for patients with other hypopigmentation disorders, including pityriasis alba and postinflammatory hypopigmentation.

Mutations in the TYR gene, *TYR,* cause oculocutaneous albinism type 1 (OCA1), an autosomal recessive disorder characterized by reduced melanin production in the hair, epidermis, and eyes (1). Importantly, patients with albinism have decreased best-corrected visual acuity and difficulty in high-glare environments. OCA1 is subdivided into 2 categories: 1) OCA1A, in which TYR activity and melanin synthesis are undetectable, and 2) OCA1B, in which TYR activity and melanin deposition are present — but reduced compared with unaffected individuals. No specific treatments are currently known for OCA1, and care is supportive.

We recently described the expression and purification of the human recombinant intramelanosomal domain of TYR (hTYR, hTYR^WT^) and OCA1B-related hypomorphic variant Pro406Leu (hTYR^P406L^) (14–16). Here, we developed a high-throughput screening (HTS) approach using the hTYR^WT^ protein to simultaneously identify its activators and inhibitors. After screening over 34,000 compounds, we identified 7 potential activators and 65 potential inhibitors (32 of which had not been previously reported). Four activators and 6 uncharacterized inhibitors were further examined with detailed enzymology. Among the potential activators, ampyrone (4-aminoantipyrine; IUPAC, 4-Amino-2,3-dimethyl-1-phenyl-3-pyrazol-5-one) increased the catalytic activity of both hTYR^WT^ and hTYR^P406L^. Ampyrone is a pyrazolone derivative belonging to the class of nonsteroidal antiinflammatory drugs (NSAIDs). As a member of this group, it contains a 5-membered heterocyclic ring with 2 nitrogen atoms and 1 oxygen atom, a structural motif characteristic of early synthetic pharmaceuticals. Pyrazolones were first introduced in the late nineteenth century, with antipyrine synthesized in 1883 by Ludwig Knorr (17). Pharmacologically, ampyrone shares key properties of the pyrazolone family, including mild and short-lived inhibition of platelet aggregation compared with aspirin (18). Unlike many traditional NSAIDs, pyrazolone derivatives are associated with relatively low gastrointestinal and renal toxicity and demonstrate a comparatively favorable safety profile even at high doses. Although some compounds in this class have been withdrawn in certain countries, pyrazolone derivatives continue to be used in specific clinical contexts for the management of postoperative pain, arthritis, gout, colic, cancer-related pain, and migraine (19).

Computational analysis indicates that ampyrone enhances catalytic activity and may help restore hTYR^P406L^ by improving structural stability and reorganizing active site geometry, including copper-histidine coordination and substrate placement, resulting in a more rigid and energetically stable active site. Using an in-house developed liquid chromatography-mass spectroscopy–based (LC-MS–based) tyrosine tracing technique, we observed increased melanin synthesis in live human normal and OCA1B melanocytes within minutes of ampyrone treatment. Finally, ampyrone also increased epidermal pigmentation in a 3-dimensional (3D) skin model.

Overall, our findings suggest that ampyrone may represent a therapeutic lead compound for TYR activation in albinism and other hypopigmentation disorders, as well as for improving UV protection in individuals with fair skin.

## Results

*HTS identified potential activators and inhibitors of hTYR^WT^*. hTYR^WT^ and hTYR^P406L^ enzymes were purified and optimized for primary and/or secondary screening of small molecule activators or inhibitors (Supplemental Figures 1 and 2; supplemental material available online with this article; https://doi.org/10.1172/jci.insight.202947DS1). Both proteins were expressed in whole *Trichoplusia ni* larvae using a baculovirus system and purified as soluble proteins from crude larval lysates by immobilized metal affinity chromatography (IMAC) followed by size-exclusion chromatography (Supplemental Figure 1A). Final purification on a Superose 12 10/300 GL column yielded single monomeric peaks with an apparent molecular weight of 56.4 (hTYR^WT^) and 55.2 (hTYR^P406L^) kDa (Supplemental Figure 1B). Protein identity was confirmed by SDS-PAGE and Western blot, showing broad ~60 kDa bands consistent with N-linked protein glycosylation. Previous in vitro experiments confirmed that the hTYR^P406L^ variant retains 35% of hTYR^WT^ specific activity (15).

**Figure 1:**
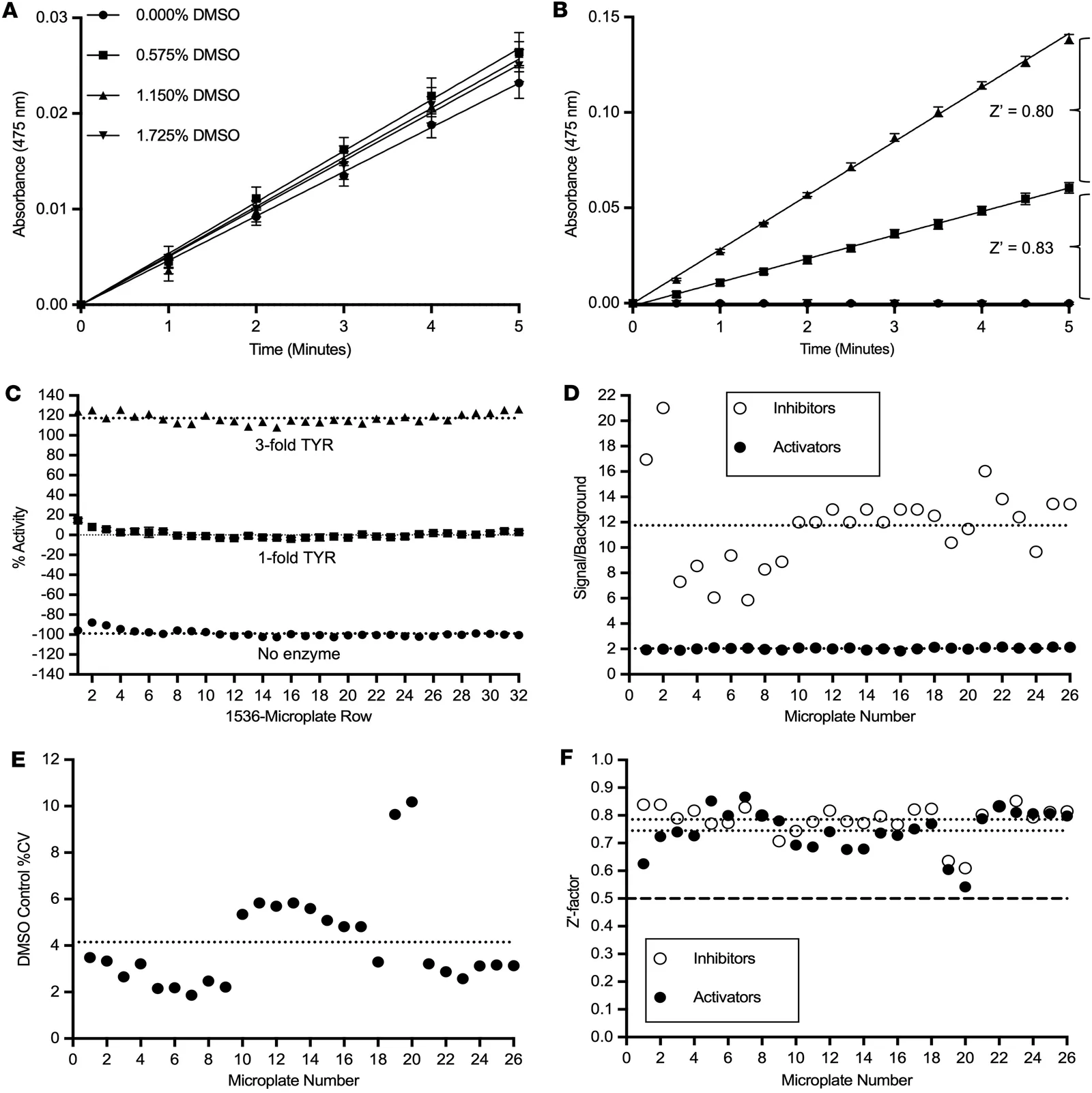
Validation and performance of a miniaturized HTS assay for simultaneously identifying both activators and inhibitors of hTYR^WT^ diphenol oxidase activity in vitro. (**A**) The optimized diphenol oxidase activity assay was performed with hTYR^WT^ (0.75 μM) in the absence and presence of increasing concentrations of the vehicle. No reduction in enzymatic activity was observed at concentrations up to 1.73%. All data show the mean ± SD (*n* = 3 technical replicates). (**B**) Kinetics of the optimized assay controls including the high activity control (triangles, 3-fold hTYR^WT^, 4.5 μM), baseline control (squares, hTYR^WT^, 1.5 μM), and low activity control (diamonds, no enzyme). Together, the controls enabled the assessment of assay performance to screen for activators (Z’ = 0.8) and inhibitors (Z’ = 0.83). All data show the mean ± SD (*n* = 3 technical replicates). (**C**) Endpoint percentage activity of the controls within a 1536-well microplate including the high activity control (*n* = 32), baseline control (*n* = 32), and low activity control (*n* = 32). (**D**) Signal/background values from the controls to identify activators (filled circles) and inhibitors (open circles) among the 26 microplates of the primary HTS. The average signal/background for the low activity control to identify inhibitors (no enzyme, open circles) was 11.75 (dotted line) and 2.0 for the high activity control to identify activators (3-fold hTYR^WT^, filled circles). (**E**) The average coefficient of variation from the DMSO-treated baseline control for 26 microplates was 4.1% (dotted line), which demonstrates low variability. (**F**) The Z’-factor was calculated from the controls of each microplate to serve as an acceptance criteria and to assess assay performance, where a Z’-factor value ≥ 0.5 (dashed line) is considered to be excellent. For the low activity control (open circles), Z’-factor values ranged between 0.61 and 0.85, with an average of 0.79 (dotted line) among 26 microplates. For the high activity control (filled circles), Z’-factor values ranged between 0.54 and 0.87, with an average of 0.74 (dotted line) among 26 microplates.

hTYR catalyzes the oxidation of mono- and diphenols (L-tyrosine and L-DOPA, respectively) to their corresponding quinones, which, in turn, produce dopachrome, an orange/brown product that absorbs light at 475 nm and can be measured spectrophotometrically. We developed a miniaturized 1,536-well kinetic assay for hTYR^WT^ diphenol oxidase activity that was optimized to identify activators and inhibitors from chemical libraries, unaffected by vehicle, and exhibited Z’-factor values ≥ 0.8 (Figure 1, A–C). A primary HTS was conducted on 34,051 compounds with a 4% average coefficient of variation for the vehicle-treated baseline control and an average Z’-factor of 0.74 (activator) and 0.79 (inhibitor) (Figure 1, D–F, and Supplemental Figure 3A).

Among the hits from the primary screen, 115 compounds with inhibitory activity were identified. Of these, 6 top-performing candidates were selected for evaluation in a confirmatory in vitro assay (Supplemental Figure 3, B and C, and Supplemental Table 1). The remaining compounds were excluded from further analysis because they were previously characterized TYR inhibitors, established topical agents or antibiotics, or associated with known toxicity, carcinogenicity, or other unfavorable safety profiles. The diphenol oxidase activity of hTYR^WT^ was inhibited in a concentration-dependent manner by all 6 compounds — 4-anilinophenol; idronoxil; anethole trithione; pestanal (fenaminosulf); 3’,4’-dihydroxyflavone; and 6-thioguanine — with IC_50_ values of 3.20, 29.13, 1.45, 8.12, 9.22, and 540 μM, respectively (Supplemental Figure 4A).

In total, 9 compounds were identified as potential activators, of which 4 were confirmed using the same secondary in vitro assay applied for inhibitors (Supplemental Figure 3B, C, and Supplemental Table 1). Of the other 9 potential activators, 5 were excluded, including phloretin and brazilin due to prior characterization as TYR inhibitors (20, 21); 4,4’-Thiodianiline because of its known carcinogenicity; and the 2 Genesis library hits that were inactive in the secondary assay. Of the 4 hits confirmed by the secondary assay, hydroxytacrine maleate exhibited a modest, concentration-dependent increase in absorbance at 475 nm over time and sennoside B showed a modest and nonconcentration-dependent effect, whereas 4-dimethylaminoantipyrine had no effect at any of the tested concentrations (Supplemental Figure 4B). In contrast, a significant increase in hTYR^WT^ diphenol oxidase activity was observed in the presence of ampyrone (Supplemental Figure 4C).

### Ampyrone increases the enzymatic activity of hTYRWT and hTYRP406L

**Figure 2:**
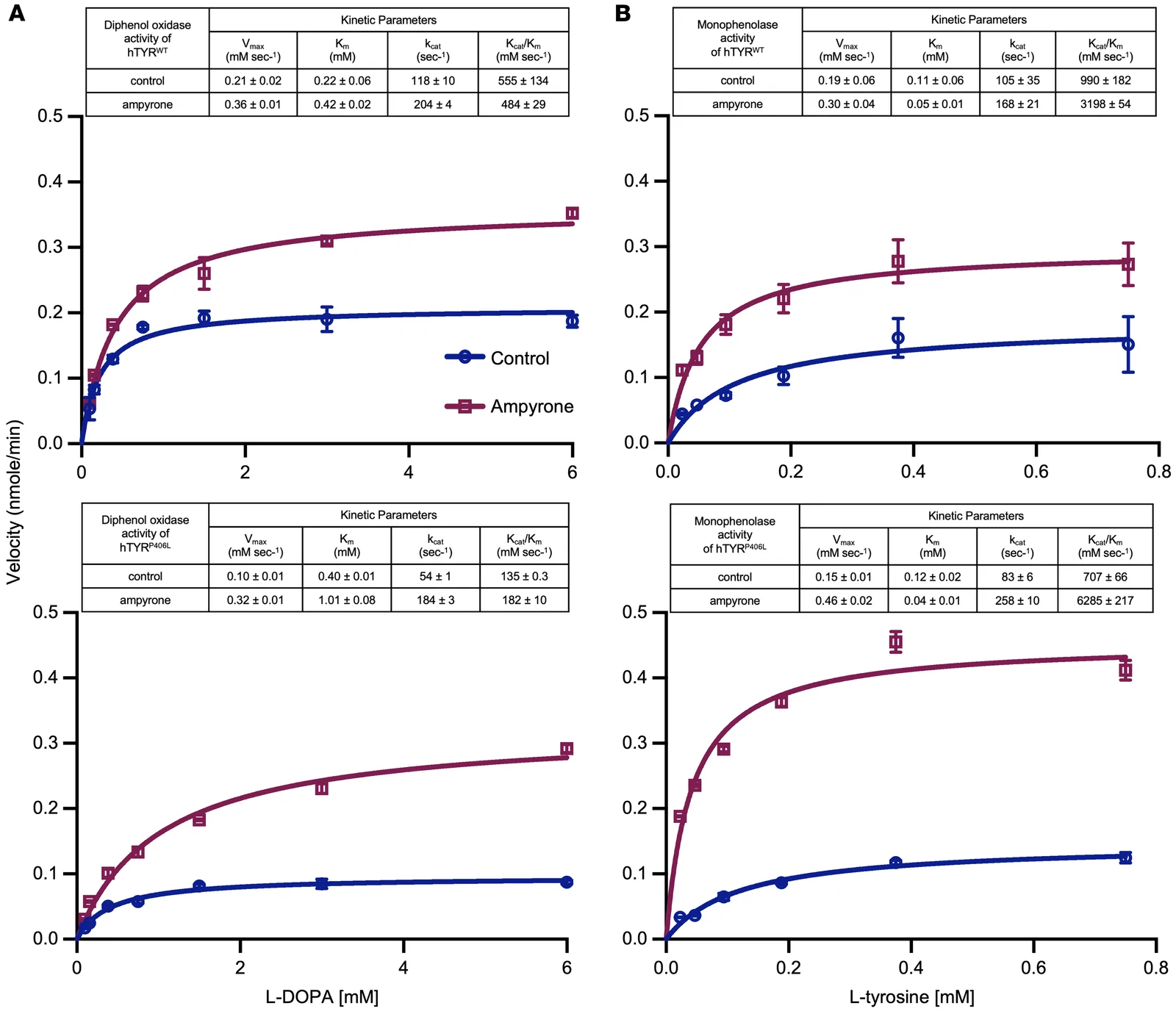
Effect of 10 mM ampyrone on the diphenol oxidase and monophenolase activities of hTYR^WT^ and hTYR^P406L^. (**A** and **B**) Michaelis-Menten plots showing diphenol oxidase (**A**) and monophenolase (**B**) activities of hTYR^WT^ (top panels) and hTYR^P406L^ (bottom panels), measured at 37°C in the presence (red) or absence (control, blue) of 10 mM ampyrone using increasing concentrations of L-DOPA or L-tyrosine, respectively. Curves represent nonlinear regression fits to the Michaelis-Menten equation generated in GraphPad Prism 10. Insets display the corresponding kinetic parameters for both diphenol oxidase and monophenolase activities of hTYR^WT^ and hTYR^P406L^. Data are presented as mean ± SD from 3 independent experiments.

Ampyrone demonstrated a concentration-dependent increase in diphenol oxidase activity of both hTYR^WT^ and hTYR^P406L^ enzymes, with EC_50_ values of 0.67 and 3.57 mM, respectively (Supplemental Figure 4C, D). Incubation with 10 mM ampyrone increased V_max_, K_m_, and k_cat_ for both enzymes (Figure 2A and Supplemental Figure 5A). In hTYR^WT^, k_cat_ increased by 73%; however, the concurrent 91% increase in K_m_ resulted in a modest 15% decrease in catalytic efficiency (k_cat_/K_m_), indicating that the enhanced turnover did not fully compensate for the reduction in apparent substrate affinity. In contrast, in hTYR^P406L^, ampyrone increased k_cat_ by 238% and K_m_ by 153%, yielding a net 35% increase in catalytic efficiency. Thus, although substrate affinity decreased for both enzymes in the presence of ampyrone during diphenol oxidase activity, the pronounced increase in turnover led to improved overall efficiency in the mutant and only a modest reduction in the WT enzyme.

Further kinetic analyses showed that ampyrone had a substantially stronger effect on monophenolase activity (Figure 2B and Supplemental Figure 5B). In hTYR^WT^, ampyrone increased V_max_ by 58%, decreased K_m_ by 55%, and enhanced k_cat_ by 60%, resulting in a 223% increase in catalytic efficiency. The effect was even more pronounced in hTYR^P406L^, where ampyrone increased V_max_ by 207%, decreased K_m_ by 67%, and elevated k_cat_ by 209%, leading to a 788% increase in catalytic efficiency. These results indicate that ampyrone enhances both catalytic turnover and substrate affinity in monophenolase activity, producing a marked increase in catalytic efficiency, particularly in the P406L mutant. Together, these findings demonstrate a substantially greater enhancement of monophenolase catalytic efficiency compared with diphenol oxidase activity, especially in the P406L variant.

#### Modeling of ampyrone effects on hTYR.

**Figure 3:**
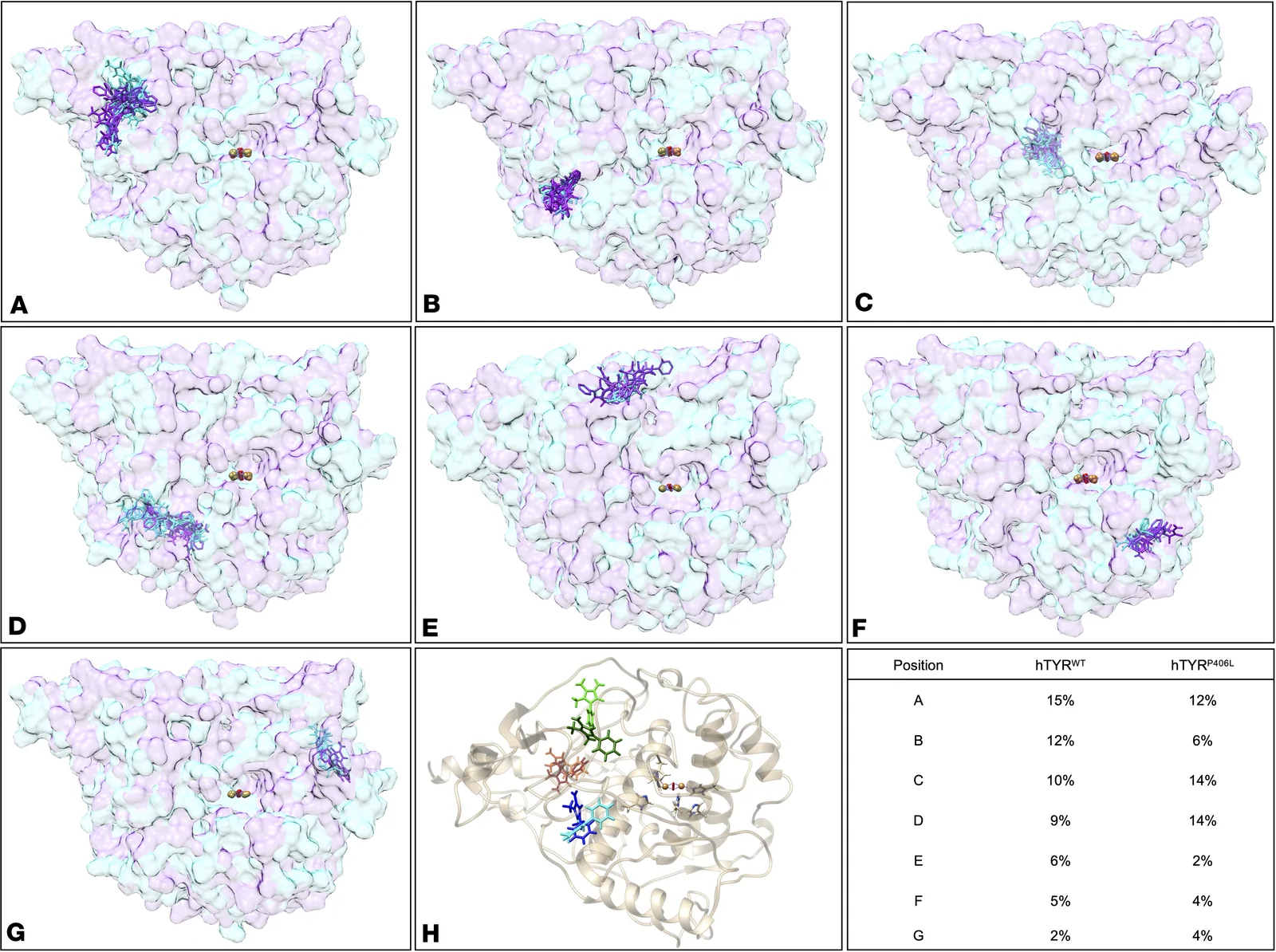
The most frequently occupied ampyrone binding positions outside the active site are similar for both hTYR^WT^ and hTYR^P406L^, as determined by molecular docking. (**A**–**G**) Ampyrone docking poses are shown in purple for hTYR^WT^ and cyan for the hTYR^P406L^. Transparent purple represents the surface of the superimposed hTYR^WT^, while cyan indicates the hTYR^P406L^. The copper atoms: CuA (left) and CuB (right) in the active sites are depicted as orange spheres. (**H**) The top 3 binding sites for ampyrone in hTYR^WT^ (Position A/light green, Position B/cyan, and Position C/orange) and hTYR^P406L^ (Position C/red, Position D/royal blue, Position A/dark green). The table summarizes the binding positions and their occupancy frequency.

One hindrance to understanding hTYR enzymology is the lack of a TYR x-ray crystal structure. Therefore, to examine how ampyrone binding leads to an increase in enzyme activity, we performed molecular modeling of hTYR based on the crystal structure of the closely related enzyme hTYRP1 (22). Ampyrone was docked to models of both the hTYR^WT^ and hTYR^P406L^. Several binding positions outside the active site were shared between the 2 proteins (Figure 3). The top 3 binding sites were selected for further molecular dynamics (MD) simulations. In both enzyme variants, 1 ampyrone molecule consistently bound near residues P205-T226. A second molecule bound at the interface between the signal peptide, the 150–170 loop, and the β-sheet 200 region. The third binding site differed between the 2 variants: in the hTYR^WT^ model, ampyrone bound to the loop connecting β-sheets within the Cys-rich subdomain, whereas in the hTYR^P406L^ model, it occupied a cavity separating the Cys-rich and catalytic subdomains.

We next sought to understand how ampyrone binding affects the conformation and flexibility of hTYR residues using MD simulations. Ampyrone binding sites and representative individual trajectories are presented in Supplemental Figure 6. Based on triplicate simulations, the root-mean square deviation (RMSD) and the root mean-square fluctuation (RMSF) were calculated (Supplemental Figure 7). RMSD provides a measure of overall protein stability over time, whereas RMSF reflects the average positional fluctuation of individual residues, offering insight into the local flexibility of the polypeptide chain. Ampyrone binding had minimal effect on the RMSD profile of hTYR^WT^ but notably reduced the elevated RMSD observed in hTYR^P406L^, bringing it closer to the WT profile (Supplemental Figure 7A). Similarly, RMSF analysis revealed reduced movement upon ampyrone binding to both hTYR^WT^ and hTYR^P406L^, with the latter displaying a more pronounced effect (Supplemental Figure 7B). However, ampyrone did not restore coordinated motions within helices I172-S184 and P205-T226, as reflected by the lack of recovery in dynamical cross-correlation matrix (DCCM) patterns, which quantify how pairs of residues move in a correlated or anticorrelated manner throughout MD simulations (Supplemental Figure 8).

**Figure 4:**
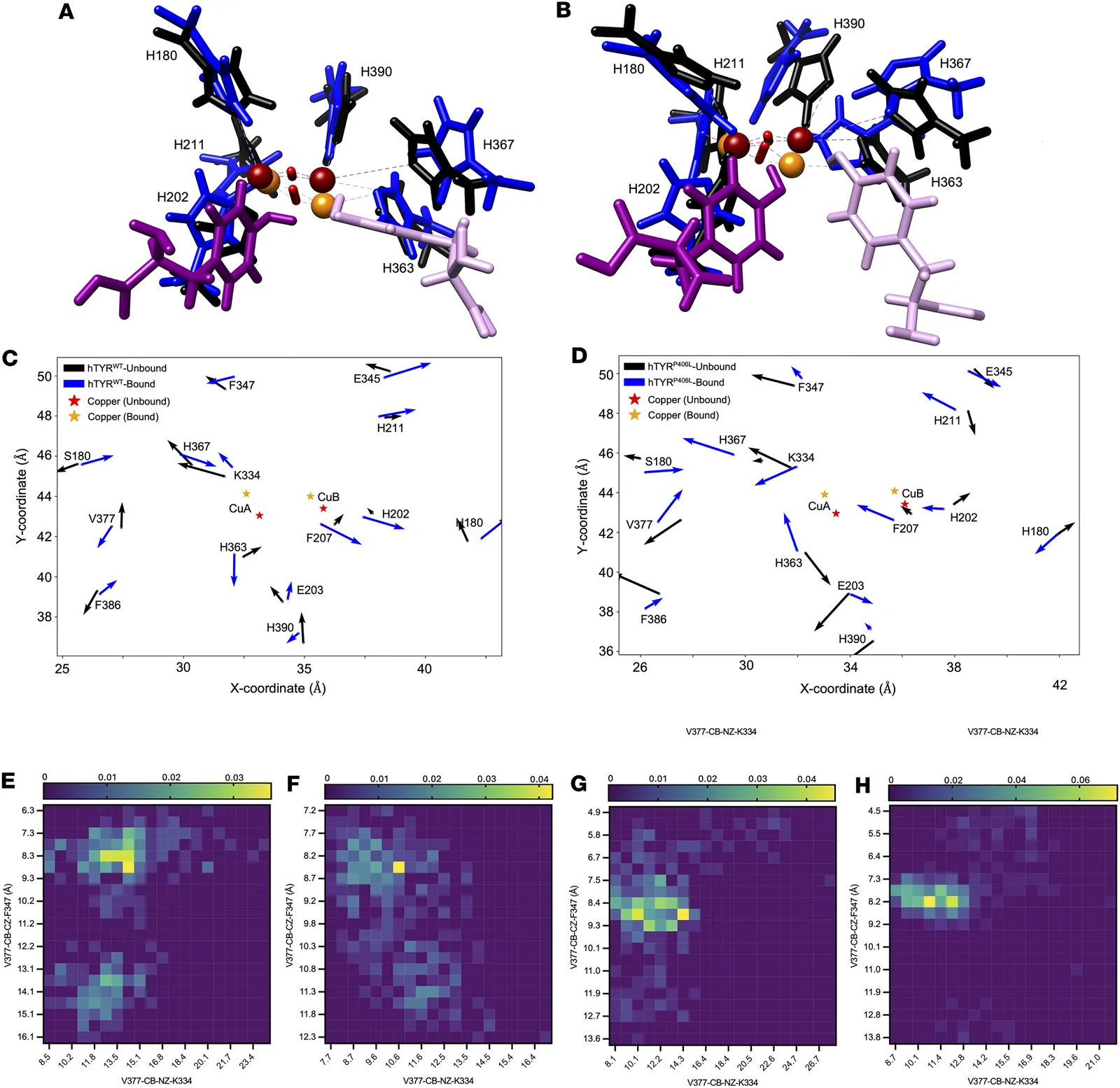
Computational modeling of ampyrone effects in hTYR^WT^ and hTYR^P406L^. (**A** and **B**) The active site in the absence (Protein/Black, Substrate/Light purple, Cu/Red, O_2_/Red) and presence (Protein/Blue, Substrate/Dark purple, Cu/Orange, O_2_/Red) of ampyrone shows movements in the 6 catalytic histidine residues for both hTYR^WT^ (**A**) and hTYR^P406L^ (**B**) when binding L-tyrosine. Coppers A and B (CuA and CuB) can be read left to right in all panels. (**C** and **D**) Porcupine plots display the movements of the α carbon atoms of select active site residues in the absence and presence of ampyrone for hTYR^WT^ (**C**) and hTYR^P406L^ (**D**). (**E**–**H**) Free energy landscapes mapping the distance V377-CB-CZ-F347 and V377-CB-NZ-K334 in angstroms (Å) for hTYR^WT^-unbound (**E**), hTYR^WT^-bound (**F**), hTYR^P406L^-unbound (**G**), hTYR^P406L^-bound (**H**).

Additionally, the presence of ampyrone resulted in the rearrangement of active site residues in hTYR, including the 6 copper-coordinating histidine residues, affecting the binding position of L-tyrosine (Figure 4, A and B). Distances and coordination angles between copper atoms and H202, H211, and H363 shifted substantially and consistently for both hTYR^WT^ and hTYR^P406L^ (Supplemental Figures 9 and 10). These structural perturbations in the presence of ampyrone were further demonstrated by porcupine plots, which illustrate both the direction and extent of residue movements along the dominant motion pathway during the simulation. These plots showed increased mobility of residues near copper sites (e.g., F207, H202, E203), while more distal residues remained relatively stable (Figure 4, C and D). The direction of movement, however, appears to be residue specific. To assess how ampyrone affects active site conformational dynamics, we performed free energy landscape (FEL) analysis using distances between Cß atoms of V377 and the side chains of 2 catalytically important residues, F347 (CZ) and K334 (NZ), as reaction coordinates. Ampyrone binding reduced conformational heterogeneity in both hTYR^WT^ and hTYR^P406L^, as shown by narrower and deeper energy minima, indicating a more rigid and energetically stabilized active site (Figure 4, E–H). Modeling predictions that ampyrone improves hTYR protein stability were experimentally confirmed using a urea denaturation approach (Supplemental Figure 11).

#### Ampyrone increases eumelanin production in human normal and OCA1B melanocytes.

**Figure 5:**
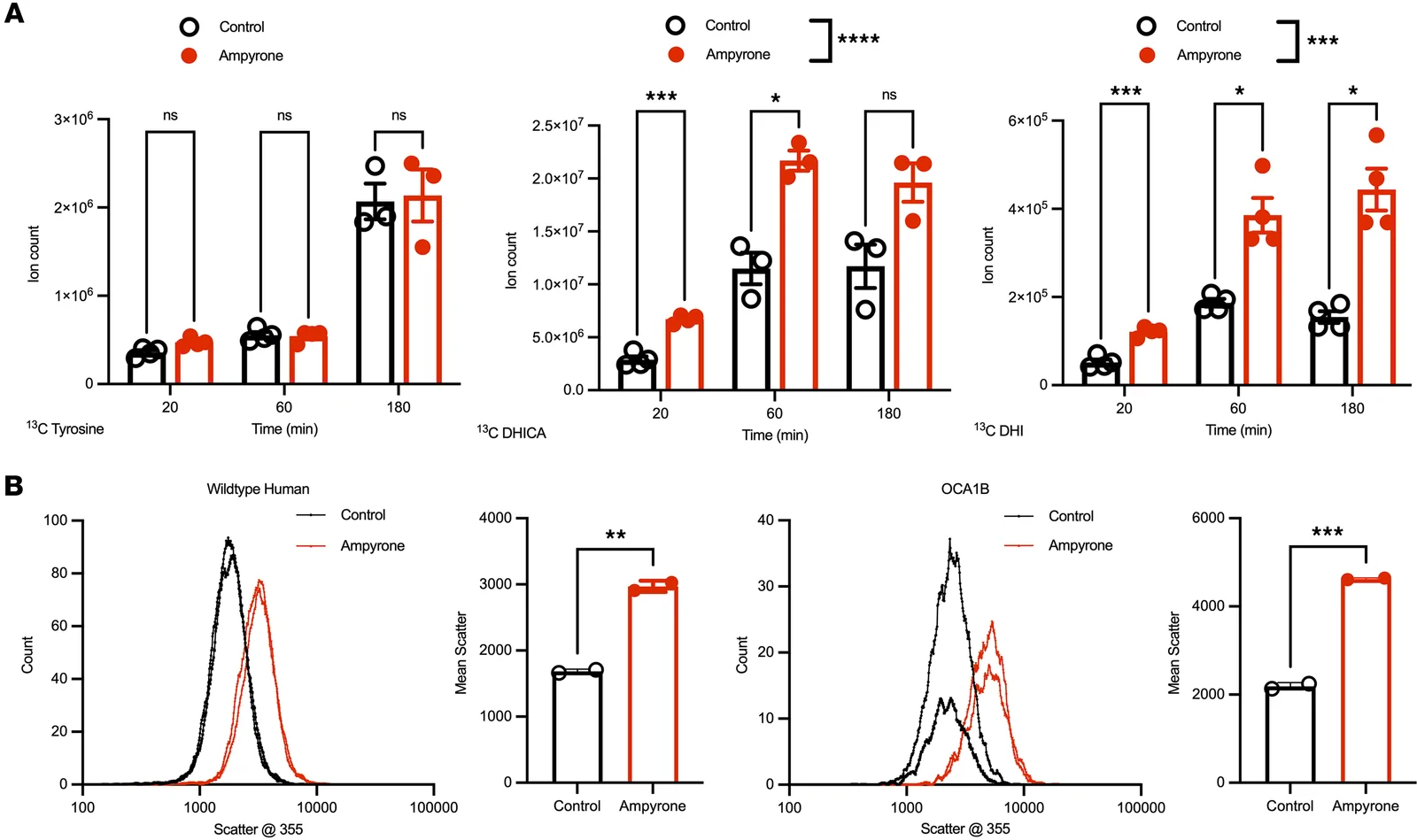
Ampyrone induces melanin synthesis in melanocytes. (**A**) LC-MS tracing of ^13^C-tyrosine metabolism in the absence (black) or presence of ampyrone (2 mM, red). Ion counts detected over time of tyrosine (left), DHICA (middle), and DHI (right). (**B**) Flow cytometry of human WT (WT Human, C4 cell line) and OCA1B (OCA1B, OCA1B-1125 cell line) melanocytes after 48 hours in vehicle (control, black) or ampyrone (0.2 mM, red). The left panels show histograms of cell counts at different amounts of scatter at 355 nm. The right panels show the mean scatter at 355 nm. Raw scatter plots from this experiment are shown in Supplemental Figure 12. (**A**) Repeated measures ANOVA with a post hoc Tukey’s test. *N* ≥ 3. (**B**) Student’s 2-tailed *t* test, unpaired (*n* ≥ 2). *P < 0.05; **P < 0.01; ***P < 0.001; ****P < 0.0001.

Traditional methods of measuring melanin rely on the accumulation of polymerized melanin and the analysis of total cellular melanin by quantifying degradation products by HPLC (23). However, this approach cannot capture immediate changes in melanin synthesis, severely limiting its application to in vivo screening of drugs that alter melanin synthesis. We recently developed an LC-MS method for measuring melanin synthesis that utilizes ^13^C-labeled tyrosine, thereby allowing for the detection of melanin synthetic intermediates over time (24). Using this method, we found that ampyrone induced an increase in the 2 main eumelanin synthetic intermediates, 5,6-dihydroxyindole-2-carboxylic acid (DHICA) and 5,6-dihydroxyindole (DHI), within 20 minutes (the earliest measurable time point) without affecting tyrosine uptake into melanocytes (Figure 5A). Ampyrone treatment induced a further increase in DHICA and DHI at 1 hour, which then plateaued at 3 hours, suggesting an equilibrium between new synthesis and polymerization into melanin (Figure 5A). We next examined the effect of ampyrone on melanin synthesis over multiple days in WT and OCA1B human melanocytes by measuring changes in side-scatter using flow cytometry and found that ampyrone treatment for 48 hours increased melanin synthesis (Figure 5B and Supplemental Figure 12, A and B). In each case, these human melanocytes carry a TYR pathogenic allele (p.Arg217Trp or p.Pro31Leu) over the most common pathogenic OCA1B allele (–301C, 575C>A [Ser192Tyr]; 1205G>A[p.Arg402Gln]) (25, 26). OCA type 2 is caused by decreased activity of WT TYR due to an abnormally acidic melanosome pH (27). We next asked if ampyrone affected melanin synthesis in OCA2 mouse melanocytes. Treatment of mouse OCA2 melanocytes with ampyrone for 48 hours also increased melanin levels as measured by increased side-scatter (Supplemental Figure 12C). These findings indicate that ampyrone treatment may be an effective approach for increasing melanin synthesis in human melanocytes with both WT and defective TYR activity (28, 29).

#### Ampyrone increases pigmentation in a human 3D epidermal model.

**Figure 6:**
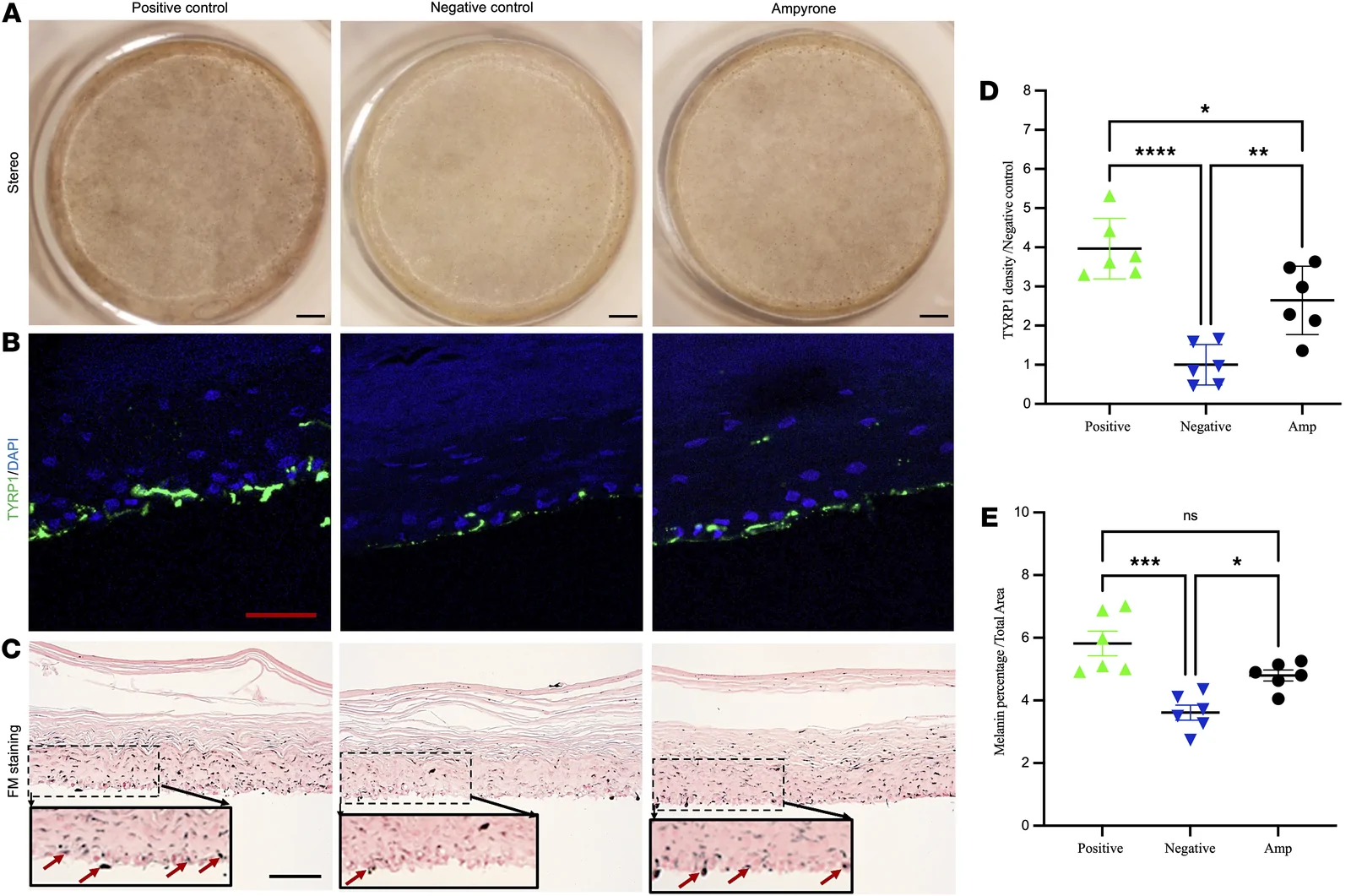
Ampyrone treatment enhances pigmentation in 3D human skin culture. (**A**) Stereo microscopic (Stereo [10×] images, represents positive, negative, and ampyrone-treated (200 μM) samples). (**B**) TYRP1 expression was detected by immunofluorescence using anti-TYRP1 (green) with DAPI nuclear counterstain (blue). (**C**) The Fontana-Masson (FM) staining examines the effects of ampyrone on epidermal melanin synthesis. The inset highlights aggregated melanin granules, marked by red arrows. (**D**) Quantification of TYRP1 expression was analyzed as the percentage of mean relative green density area normalized to the negative control. (**E**) Quantitation of FM staining (melanin) expressed as a percentage across the area of basal and spinous layer. *n* = duplicates, 3 replicates. ANOVA post hoc Tukey’s test. *P < 0.05; **P < 0.01; ***P < 0.001; ****P < 0.0001. Scale bar: 1000 μm (**A**), 50 μm (**B**), 100 μm (**C**). These data are representative of an experiment repeated 3 times.

Skin pigmentation is primarily driven by complex interactions between melanocytes and keratinocytes, the principal cellular components of the epidermis. To model human skin, we used the MelanoDerm 3D epidermal culture system consisting of cocultured normal, human-derived epidermal keratinocytes (NHEK) and normal, human-derived melanocytes (NHM), which is widely used to study epidermal pigmentation in vitro (30, 31). Ampyrone was applied every other day for 21 days. Macroscopic imaging showed gradual epidermal darkening, and the pigmentation produced by ampyrone was substantially higher than the untreated control (Figure 6A). As a positive control, the 3D culture epidermal system was treated with α-melanocyte stimulating hormone (α-MSH)/β-fibroblast growth factor (β-FGF). Interestingly, ampyrone induced visual pigmentation to approximately 50% of α-MSH/β-FGF treatment (Figure 6A, Supplemental Figure 13A). To assess melanogenesis in the 3D culture epidermal system, we examined TYRP1 expression by immunofluorescence staining. Strong TYRP1 signals were observed in the positive control, whereas the untreated negative control showed only modest expression. The ampyrone-treated 3D cultures exhibited increased TYRP1 intensity compared with the negative control, indicating that ampyrone has some effect on melanogenesis (Figure 6, B and D). Furthermore, to assess melanin synthesis and distribution, we performed Fontana-Masson (FM) staining. Quantitative analysis of melanin-positive areas in the basal layer and spinous layer showed that α-MSH/β-FGF induced extensive melanin deposition, while the negative control had minimal melanin. Treatment with ampyrone led to substantial FM staining within the epidermis with melanin mainly clustered basal layer (Figure 6C). Overall FM staining was markedly higher compared with the negative control rising to almost to the same level as α-MSH/β-FGF (Figure 6E).

We also monitored melanocyte cellular morphology in our 3D culture system, and ampyrone treatment consistently exhibited multiple densely elongated dendrites interdigitating between keratinocytes. The level of dendricity was intermediate between positive and negative controls (Supplemental Figure 13A). H&E staining revealed that, under all conditions, tissue morphology was well preserved, showing an intact epidermal layer and normal cellular organization (Supplemental Figure 13B). Furthermore, cell apoptosis detected by TUNEL staining revealed no evidence of apoptosis (Supplemental Figure 13C). Taken together, these data suggest that ampyrone increases epidermal pigmentation and melanogenesis without evidence of toxicity.

## Discussion

Pharmacologic activation of human TYR represents a promising but underexplored strategy for enhancing melanin synthesis in disorders of hypopigmentation. In this study, we developed a HTS platform based on recombinant hTYR and identified multiple inhibitors and activators, including ampyrone as a small-molecule activator of both hTYR^WT^ and the disease-associated hTYR^P406L^ variant (Supplemental Table 1). In parallel, we demonstrated that LC-MS–based ^13^C-tyrosine tracing enables rapid and sensitive detection of changes in melanin synthesis in a cell-based system, overcoming the limitations of conventional assays that are influenced by high background melanin levels. Together, these approaches establish a framework for identifying direct regulators of TYR activity and provide a foundation for pharmacological modulation of melanogenesis.

To further define the functional consequences of TYR activation, we focused our efforts on characterizing the effects of ampyrone on hTYR^WT^ and the common OCA1B-associated hTYR variant P406L. Ampyrone belongs to a pharmacologically versatile chemical class whose derivatives exhibit a wide range of biological activities, including antiinflammatory, analgesic, antimicrobial, anticancer, and antioxidant effects (32–36). Moreover, ampyrone-based analogues have been extensively investigated as acetylcholinesterase and butyrylcholinesterase inhibitors for Alzheimer’s disease therapy, highlighting the adaptability of this chemical framework (37–40). Importantly, compounds structurally related to ampyrone are already approved for human use, which may facilitate its translation into human trials. We found that ampyrone differentially modulates the 2 catalytic activities of TYR, with a markedly stronger effect on monophenolase activity. In this mode, ampyrone enhances both substrate affinity and turnover, resulting in a substantial increase in catalytic efficiency in hTYR^WT^ and, more prominently, in hTYR^P406L^. In contrast, diphenol oxidase activity shows a more nuanced response, with increased turnover accompanied by reduced apparent substrate affinity, leading to modest or reaction-dependent changes in efficiency. These findings indicate that ampyrone preferentially enhances the physiologically initiating and rate-limiting step of melanogenesis and exhibits pronounced rescue effects in the disease-associated mutant. We note that our human OCA1B cultured melanocytes harbor a distinct disease-causing allele (-301, 575C>A [Ser192Tyr]; 1205G>A[p.Arg402Gln]), suggesting that this stimulation of TYR activity is not unique to the hTYR^P406L^ allele.

Ampyrone treatment showed no overt toxicity in human melanocytes in culture or in human 3D skin model cultures, even after 3 weeks of exposure, suggesting that it could serve as a lead compound for the development of safe and effective TYR agonists. MD simulations predict that ampyrone transiently engages multiple surface pockets in both hTYR^WT^ and hTYR^P406L^, including regions outside the active site such as the P205-T226 segment and the β-sheet 200 region. Importantly, the binding areas are largely similar in both variants; differences are observed primarily in the frequency and stability of occupancy rather than in distinct structural sites. These regions correspond to flexible segments associated with coordinated movements within the core α-helical bundle (41), suggesting that ampyrone interacts preferentially with dynamically active areas of the protein. In both enzymes, ampyrone reduces residue fluctuations, particularly at the junction of the cysteine-rich and catalytic subdomains, consistent with a stabilizing effect that is more pronounced in the P406L variant.

Ampyrone also promotes subtle rearrangements of catalytic residues, including changes in copper-coordinating histidine geometry, and reduces conformational flexibility in residues implicated in substrate accommodation (42). Although these findings are derived from computational analyses and require experimental validation, the combined modeling and kinetic data support a mechanism in which ampyrone increases the frequency of catalytically competent conformations rather than binding to a unique active site pocket. A direct causal relationship between distal pocket occupancy and catalytic enhancement remains to be established; future targeted mutagenesis or domain-specific perturbation studies will be necessary to confirm their functional relevance and to guide rational optimization of derivative compounds.

We next employed an in vivo–like 3D human skin model to evaluate the compound’s effects in a more physiologically relevant system. MelanoDerm is a well-differentiated, 3D organotypic epidermal model that incorporates human melanocytes and recapitulates key features of human epidermal structure and melanocyte-keratinocyte interactions, making it well suited for in vivo–like evaluation of pigmentation-enhancing compounds. Over 21 days of treatment, ampyrone markedly increased pigmentation at macroscopic, tissue, and cellular levels compared with the untreated negative control, while almost reaching similar levels of pigmentation as the α-MSH/β-FGF positive control. At the molecular level, ampyrone elevated expression of TYRP1, a melanosomal enzyme whose abundance reflects both melanogenic pathway activation and melanosome maturation. These data suggest that direct stimulation of TYR activity by ampyrone can activate melanogenesis. Ampyrone did not disrupt melanocyte number or dendritic morphology and had no effect on epidermal morphology or apoptosis, indicating that ampyrone exhibited no detectable cytotoxicity. We note that an ampyrone-based therapeutic would likely be given to patients over a longer period of time than is experimentally possible using our 3D culture system; therefore, greater, cumulative effects on melanin pigmentation remain possible. Collectively, these 3D skin model data support ampyrone as a promising TYR agonist lead compound for developing therapies for hypopigmentation disorders of the skin or eyes.

We note that the millimolar EC_50_ values for ampyrone require additional efforts to improve potency and to define improved pharmacokinetic properties. Moreover, although increased pigmentation was observed in cutaneous models, such effects cannot be presumed to extend to uveal melanocytes or RPE, the correction of which are central to improving the visual deficits associated with albinism. Importantly, our recent work using human TYR-deficient RPE models (43) demonstrates that TYR activity is essential for melanosome maturation and pigmentation in ocular tissues, supporting the mechanistic rationale for targeting TYR in this context. Nonetheless, direct evaluation of ampyrone in ocular pigment cell systems will be required to determine translational relevance. As in vivo pharmacokinetics, ocular pigmentation, and visual function were not assessed in the present study, future investigations must establish systemic exposure, tissue distribution, optimal dosing parameters, and tissue-specific efficacy as part of formal preclinical development.

In considering ampyrone or similar molecules in a clinical setting, the route of delivery becomes an important consideration for future therapeutic development. Ampyrone and its derivatives have traditionally been administered orally; however, our data suggest that topical application may represent a feasible strategy to enhance pigmentation locally while minimizing systemic exposure. Previous studies have shown that ampyrone, as an active metabolite of metamizole, can penetrate the blood-brain barrier, and that topical administration of metamizole can exert local effects within the eye (44, 45). Nevertheless, direct evidence for penetration across the blood-ocular barrier remains limited. Overall, both systemic (oral) and topical delivery strategies warrant further investigation, with particular emphasis on tissue-specific distribution and ocular accessibility.

In summary, using customized HTS and LC-MS screening approaches targeting recombinant hTYR, we identified hTYR agonists and established proof-of-concept activation and functional rescue. These findings provide a mechanistic and developmental foundation for the rational optimization of compounds aimed at addressing disorders of hypopigmentation, including OCA. While substantial translational work remains, including evaluation of long-term toxicity, pharmacologic enhancement of human pigmentation represents a promising strategy with potential implications for skin protection, improving visual function, and enhancing patient quality of life.

## Methods

### Sex as a biological variable

As this study involves purified protein and cells in culture, sex was not considered as a biological variable.

### Expression and purification of WT and P406L intramelanosomal domains

The recombinant intramelanosomal domain of WT hTYR (hTYR^WT^, residues 19–469) and the OCA1B-related P406L variant (hTYR^P406L^) were engineered with a His-tag, expressed using baculovirus, and produced in whole insect *Trichoplusia ni* larvae at Allotropic Tech LLC. The proteins were purified using IMAC with a His-Trap crude 5 mL column, followed by gel-filtration (GF) chromatography on HiPrep 26/60 Sephacryl S-300 and Superose 12 10/300 GL columns (Cytiva), as previously described (14–16). Fractions containing the protein of interest were concentrated using Amicon Ultra-15/10,000 NMWL centrifugal filter units (MilliporeSigma). Protein concentrations were determined by measuring *A*_260/280__nm_ with a NanoDrop 2000c UV–Vis spectrophotometer (Thermo Fisher Scientific). The identities of hTYR^WT^ and hTYR^P406L^ proteins were confirmed by Western blot analysis using the Anti-TYR (T311) antibody (Santa Cruz Biotechnology, sc-20035).

### Miniaturized hTYRWT diphenol oxidase activity assay and HTS

Using HTS, ~34,000 compounds from the Genesis Diverse Chemical Library (Genesis), the National Center for Advancing Translational Sciences (NCATS) Pharmacologically Active Chemical Toolbox (NPACT), the NCATS Pharmaceutical Collection (NPC), and the Natural Products Library (NPL) were screened with purified hTYR^WT^. The diphenol oxidase activity assay was performed in black, medium-binding, clear flat-bottom 1,536-well microplates (Greiner) with a final reaction volume of 8 μL. All steps were performed at room temperature. First, 6 μL of reaction buffer (50 mM sodium phosphate, 0.01% Triton X-100, pH 7.5) were dispensed into the 32 wells of column 1 (no enzyme, low activity control), 6 μL of reaction buffer containing 6 μM hTYR^WT^ (4.5 μM final) were dispensed into the 32 wells of column 2 (3-fold enzyme concentration, high activity control), and 6 μL of reaction buffer containing 2 μM hTYR^WT^ (1.5 μM final) were dispensed into the wells of columns 3–48. These solutions were dispensed with a BioRaptr workstation (Beckman Coulter). Next, 92 nL of dimethylsulfoxide (DMSO, vehicle control, columns 1–4) or library compounds (columns 5–48) were pin-transferred (Kalypsis) using a dip factor of 4. For the primary screen, library compounds from 10 mM stock solutions were tested at a final concentration of 115 μM. Following the transfer of vehicle or test compounds, the microplates were spun at 377*g* for 1 minute and allowed to incubate for 30 minutes. Before the addition of substrate, absorbance was measured at 475 nm with a ViewLux uHTS microplate imager (PerkinElmer) to quantify any compound-related absorbance that was independent of hTYR^WT^ diphenol oxidase activity. The exposure time was 1 second with an excitation energy of 4,000, a readout speed of 10 μs, a 50X readout gain, and 2X image binning. The absorbance protocol included a 480 nm ± 10 nm (480/20) narrow band interference excitation filter and a clear infrared damping 400–750 nm emission filter. The hTYR^WT^ diphenol oxidase reaction was initiated by the addition of 2 μL of reaction buffer containing 1.72 mM L-DOPA (Sigma-Aldrich) (430 μM final) to all wells in columns 1–48. Immediately following substrate addition, the microplates were spun at 377*g* for 1 minute, and absorbance was measured at 30-second intervals for 20 minutes. Activity was measured as delta by comparing the absorbance at a final time point with that of the initial time point.

### TYR activation assays

The diphenol oxidase activities of hTYR^WT^ and hTYR^P406L^ enzymes were measured spectrophotometrically using a SpectraMax i3 multi-mode detection platform, with data analyzed by SoftMax Pro software (version 6.5, Molecular Devices). Enzymes were incubated at 37°C with 1.5 mM L-DOPA (MilliporeSigma) in the absence or presence of ampyrone (MilliporeSigma), and catalytic activities were monitored over 10 hours by measuring dopachrome formation at 475 nm (ε_dopachrome_ = 3,700M^–1^ cm^–1^). For the measurement of hTYR^WT^ activity during unfolding, the enzyme was incubated with urea at concentrations ranging from 0M to 8M for 1 hour, and activity was determined using 1.5 mM L-DOPA as a substrate by measuring dopachrome formation at 475 nm in absence or presence of 5 mM ampyrone.

To evaluate the potency of additional small-molecule modulators, hTYR^WT^ was incubated with selected potential inhibitors at compound-specific concentrations ranges, selected based on preliminary activity screening. Enzymatic activity was measured at 37°C by recording absorbance at 475 nm every 1 minute for 120 minutes. Representative time curves were generated for each compound to monitor kinetic effects. For IC_50_ determination, endpoint absorbance values at 120 minutes were used to calculate residual enzymatic activity across the tested concentration range. Concentration-response curves were fitted using a 4-parameter nonlinear regression model in GraphPad Prism, version 10.4.0 (GraphPad Software, LLC), and IC_50_ values were calculated accordingly.

### Michaelis-Menten and Lineweaver-Burk kinetic analysis

The diphenol oxidase and monophenolase reaction rates (V) of hTYR^WT^ and hTYR^P406L^ in the presence or absence of ampyrone were determined using L-DOPA or L-tyrosine as a substrate at concentrations ranging from 0.098 mM to 6 mM or 0.023 mM to 0.75 mM, respectively. All assays were performed at 37°C in 10 mM sodium phosphate buffer, pH 7.4. Absorbance was measured at 475 nm using the SpectraMax i3 multi-mode detection platform (Molecular Devices). The Michaelis-Menten constant (*K*_m_) and maximal velocity (V_max_) were calculated from Michaelis-Menten plots using GraphPad Prism, version 10.4.0 software. The enzyme turnover rate, k_cat_, was determined as V_max_/E_t_, where E_t_ represents the protein concentration (10 nM). Lineweaver-Burk (double-reciprocal) plots were generated GraphPad Prism by plotting 1/v against 1/[L-DOPA] or 1/[L-tyrosine]. In these plots, *y*-intercept corresponds to 1/V_max_ and the *x*-intercept to –1/ *K*_m._

### Computational analysis

#### Model Preparation and MD.

A glycosylated homology model of hTYR^WT^ was previously generated using the NEI Data Commons Ocular Proteomes TYRP1 atomic model (PDB:5M8L, https://neicommons.nei.nih.gov/#/proteomeData) (46) The hTYR^P406L^ variant was created from this model using the Edit > Swap > Residue function in YASARA Structure, version 25.1.13. Both the hTYR^WT^ and hTYR^P406L^ models were energy minimized using the YASARA Options > Choose experiment > Energy minimization function. The 3D conformer structure of ampyrone was obtained from the PubChem compound database in SDF format and then converted to PDB format in UCSF Chimera, version 1.19. For the ampyrone docking experiments, which were carried out using the VINA software implemented in YASARA Structure, 10 time frames ranging from 0 ns to 10 ns were selected from the MD simulation for hTYR^WT^ and hTYR^P406L^ models. Both hTYR^WT^ and hTYR^P406L^, with and without ampyrone, underwent triplicate 100 ns MD simulations (MD_run.mcr) using the AMBER14 force field (total of 12 simulations), with snapshots saved every 1 ns. Simulations were performed at 298 K, pH 7.4, and 0.9% NaCl in a cubic cell extending 10 Å beyond the protein (94.6 Å × 94.6 Å × 94.6 Å). Initial atomic velocities were varied by altering the Randomized Seed for each simulation.

#### Principal component analysis (PCA).

Concatenated.xtc trajectory files were aligned to a reference structure, and α carbon coordinates for all 449 atoms were extracted and inputted into GraphPad Prism, version 10.4.1. PCA was performed with data standardization and parallel analysis, in accordance with Prism’s guidelines. Loadings |x| ≥ 0.7 for PC1 and PC2 subspaces were projected onto the corresponding PDB structures.

#### Molecular docking.

L-tyrosine and L-DOPA were procured from PubChem and docked to 100 ns protein structures for all 4 protein environments in YASARA Structure using the “dock_run.mcr” macro and VINA. Docking was restricted to a 20 Å^3^ box centered around the copper ions. For each ligand, 25 docking runs were performed, and the top 3 binding poses were selected based on orientation within 4 Å of the copper atoms and hydroxyl groups oriented toward the active site.

#### Visualization, RMSD, RMSF, and SASA calculations.

Structural alignments and visualizations were performed in UCSF Chimera, version 1.18.0. The superposition of hTYR^WT^ and hTYR^P406L^ models after MD simulation used the Tools > Structure Comparison > Matchmaker tool. Distances between atoms were calculated across trajectories using the Graphics > Labels > Bonds > Graph > Save tool in the Visual MD program, VMD, version 2.0.0. The RMSD and the RMSF for each trajectory were calculated in VMD by aligning frames to the 0 ns protein structure using the Trajectory > Align. The RMSF values were averaged using a TCL file in VMD’s TK Console. RMSD was calculated using the RMSD-Trajectory > Align > RMSD. Solvent-accessible surface area (SASA) for the active site was calculated in YASARA using the Analyze > Surface Area of > Object for the 100 ns protein structure for hTYR^WT^ and hTYR^P406L^ with and without ampyrone. ΔSASA was determined as the difference between bound and unbound forms for each protein.

#### DCCM and porcupine dynamic analysis.

Python, version 3.13, was used to generate a DCCM from aligned α carbon coordinates. The correlation matrix was calculated as normalized dot products (range –1 to +1). A 2D porcupine plot was created using principal component eigenvectors from GraphPad Prism, version 10.4.1, to the direction and magnitude of each vector for select active site residues.

#### FEL.

FEL were created using the Grossman weighted histogram analysis method (WHAM) for each protein with and without ampyrone to compare the highest probability conformations of the binding site. Reaction coordinates were defined by distances between gate-keeping residues: K334, F347, and V377, calculated using VMD Graphics > Labels > Bonds > Graph > Save. WHAM was run with 21 bins, tolerance of 1 × 10^−5^, 0 spring constant, 298 K temperature, and no periodic boundary padding. The minimum and maximum bins were determined by the corresponding values for each of the data sets.

### Mouse and human melanocyte culture

Mouse melanocytes (melan-ink4a^–/–^) were obtained from the Wellcome Trust Functional Genomics Cell Bank (St. George’s, University of London, London, United Kingdom), tested, and authenticated. OCA2 mouse melanocytes were generated by CRISPR KO as previously reported (47). Melan-ink4a^–/–^ and OCA2 melanocytes were cultured at 37°C and 10% CO_2_ in a Thermo Scientific Heracell Vios 160i incubator in Roswell Park Memorial Institute (RPMI) 1640 supplemented with 10% FBS, 1% penicillin-streptomycin, 2 mM glutamine, 200 nM TPA, and 200 pM cholera toxin at 10% CO_2_. NBMEL 1284 and C4 normal melanocytes and OCA1B-1235 (TYR c.649C>T, p.Arg217Trp and c.-301C;575C>A;1205G>A]) and OCA1B-1125 (TYR c.242C>T, p.Pro81Leu and c.[–301C;575C>A;1205G>A]) human melanocytes were obtained from the Biospecimen Core of the Yale Specialized Programs of Research Excellence (SPORE) in Skin Cancer (New Haven), Harvard Medical School, and the NIH, respectively. Cells were cultured at 37°C and 5% CO_2_ in a Thermo Scientific Heracell Vios 160i incubator in Ham’s F-10 (Gibco) supplemented with 5% FBS (Corning), 2 mM L-Glutamine (Gibco), 250 ng/mL Amphotericin B (Gibco), 1% penicillin-streptomycin (100 Units/mL and 100 μg/mL, respectively) (Gibco), 5 ng/mL basic FGF (Peprotech), 10 ng/mL Endothelin 1 (Sigma-Aldrich), 7.5 μg/mL 3-isobutyl-1-methylxanthine (Sigma-Aldrich), 30 ng/mL cholera toxin (Sigma-Aldrich), and 3.3 ng/mL Phorbol-12-myristate-13-acetate (Thermo Fisher Scientific). Media was replaced every third day. Mycoplasma testing was performed quarterly using commercial kits.

### LC-MS tyrosine tracing

Mouse melanocytes (melan-ink4a^–/–^) were plated in 6-well plates and grown to approximately 80%–90% confluence, before being incubated in [U-^13^C] tyrosine medium containing vehicle or ampyrone (2 mM) for 20, 40, and 60 minutes at 10% CO_2_. Cells were quickly washed twice with ice-cold PBS, followed by a quick ddH_2_O rinse and metabolite extraction using –70°C 80:20 methanol/water (LC-MS grade methanol, Thermo Fisher Scientific). The cell-methanol mixture was subjected to bead-beating for 45 seconds using a Tissuelyser cell disrupter (Qiagen). Extracts were centrifuged for 10 minutes at 13.2k RCF to pellet insoluble material and supernatants were transferred to clean tubes. The extraction procedure was repeated 2 additional times, and all 3 supernatants were pooled, dried in a speed-vac (Savant), and stored at –80°C until analysis. The methanol-insoluble protein pellet was solubilized in 0.2M NaOH at 95°C for 20 minutes and quantified using the BioRad DC assay. On the day of metabolite analysis, dried cell extracts were reconstituted in 70% acetonitrile at a relative protein concentration of 2.5 mg/mL, and 8 μL of this reconstituted extract was injected for LC-MS–based targeted stable isotope profiling. Cell extracts were analyzed by LC-MS as described previously (24, 47–50) using a platform composed of an Agilent Model 1290 Infinity II liquid chromatography system coupled to an Agilent 6550 iFunnel time-of-flight MS analyzer. Chromatography of metabolites utilized aqueous normal phase (ANP) chromatography on a Diamond Hydride column (Microsolv). Mobile phases consisted of: (a) 50% isopropanol, containing 0.025% acetic acid, and (b) 90% acetonitrile containing 5 mM ammonium acetate. To eliminate the interference of metal ions on chromatographic peak integrity and electrospray ionization, EDTA was added to the mobile phase at a final concentration of 6 μM. The following gradient was applied: 0–1.0 min, 0% B; 1.0–15.0 min, to 20% B; 15.0–29.0 min, 50% B; 29.1–37 min, 99% B. Raw data were analyzed using MassHunter Profinder 8.0 and MassProfiler Professional (MPP) 14.9.1 software (Agilent technologies). An in-house untargeted stable isotope tracing (USIT) workflow (50, 51) was employed to obtain all possible fates of [U-^13^C] tyrosine and quantitative information on the relative incorporation of tyrosine-derived metabolites based on the stable isotope labeling pattern. USIT used the Agilent untargeted metabolite profiling software (MassHunter Qualitative Analysis 8.0, MassProfinder 8.0 and MassProfiler Professional [MPP 14.9]) for an initial targeted identification of differentially expressed metabolites in cells grown in [U-^13^C] tyrosine-supplemented media.

### Measurement of melanin content by flow cytometry

NBMEL 1284 and C4 normal human melanocytes, OCA1B-1235 and OCA1B-1125 OCA1B human melanocytes, and OCA2 mouse melanocytes were treated with normal culture media and either control or with 0.2 mM ampyrone for 48 hours. Following treatment, cells were trypsinized and resuspended in 100 μL PBS without calcium and magnesium (Corning). Flow cytometry was performed using a BD Fortessa X20 5 laser analyzer. Data collection included forward scatter, side scatter, and scatter intensities at 355 nm. A minimum of 2,000 events were collected and analyzed per replicate sample. All analysis was performed using FCExpress (DeNovo Software). Events were gated by side scatter and forward scatter to isolate live cells prior to further processing. Differential light scattering due to melanin was captured as mean scatter intensity through a 379/28 nm bandpass filter using a 355 nm UV laser. Mean scatter was statistically analyzed using unpaired 2-tailed Student’s *t* test comparing vehicle with ampyrone treated cells.

### In vitro 3D tissue culture, treatment, and imaging

MelanoDerm (MEL-312-B; MatTek Corp.) is derived from donors, which is a human 3D epidermal culture system consisting of cocultured normal, human-derived epidermal keratinocytes (NHEK), and melanocytes (NHM). MelanoDerm was grown at the air-liquid interface in EPI-100-NMM-3 medium provided by the manufacturer; its composition is proprietary, but it does not contain stimulators of melanogenesis and is therefore a suitable negative control. Cells were cocultured on a collagen-coated membrane to form a multilayered, highly differentiated model of the human epidermis with the apical surface exposed to air and the basal surface exposed to culture medium (32). Prior to experiments, tissues were washed with 1 mL PBS. Positive control samples were treated with EPI-100-NMM-113 medium from the manufacturer, which contains α-MSH and β-FGF. The positive control shows that the tissues can darken. Ampyrone was dissolved in EPI-100-NMM-3 medium to a final concentration of 0.2 mM, which applied to MelanoDerm every other day for 21 days. The epidermal images were captured using a stereo microscope (ZEISS SteREO Discovery.V12). Melanocyte images were captured using an integrated digital inverted microscope (EVOS M5000 Imaging System, Thermo Fisher Scientific).

### Histological and immunofluorescence staining

On the twenty-first day of the experiment, samples were processed, embedded in paraffin blocks and sectioned to a thickness of 5 μm onto slides, which were stained with FM to highlight melanin pigmentation and H&E to stain for normal structure following standard methods (Histoserv Inc.). Images were captured using the inverted widefield microscope (Zeiss Definite Focus). We also prepared Frozen Tissues by OCT embedded for Cryostat Sections. Indirect immunofluorescence was performed with the following antibodies and dilutions: anti-TYRP1antibody (BioLegend, San Diego, CA, USA, 917801, 1:200), following incubated with secondary antibody and counterstained with DAPI and then mounted with a coverslip as described previously (33). Quantitative analysis of TYRP1 expression and FM stained in human 3D tissue was performed using ImageJ software (NIH). Cell apoptosis was detected by TUNEL using the Roche In Situ Cell Death Detection Kit (11684795910; Roche Applied Science) according to the manufacturer’s instructions. The positive control for apoptosis was generated by incubating fixed 3D skin sections with DNase I (500 U/mL) for 10 minutes to induce DNA strand breaks prior to the labeling procedure, and counterstained with DAPI.

### Statistics

Differences between tested groups were analyzed using ANOVA and student’s *t* tests, as appropriate; details are given in the text and legends of the paper.

### Study approval

As no animal or human data appear in this manuscript, no approvals were obtained.

### Data availability

All cell lines, drugs used, antibodies, and other reagents used in the study are freely available from commercial sources or from publicly available biorepositories as detailed in the manuscript. All data are compiled in the Supporting Data Values file. Human TYR cDNA plasmids will be provided upon request.

## Author contributions

MBD, YVS, NPC, JHZ, and BPB designed the experiments. MBD, ST, ZE, SJG, JB, YW, and NPC generated the figures. VKK, YJ, RK, DS, RPA, CD, CS, TC performed supporting experiments and data analysis. MDH, MB, and MS helped with screening assay design. NA ran the LC-MS samples and provided QC for the data prior to ZE doing the analysis QC, SSG, DRA, and SKL generated critical reagents or assisted with LC-MS experiments. MBD, JHZ, and BPB wrote the manuscript with all authors providing feedback.

## Conflict of interest

JHZ is a paid consultant and on the medical advisory board of Hoth Therapeutics and AmorePacific. JHZ is a paid consultant for Tanabe Pharmaceuticals. JHZ is a paid consultant and receives sponsored research funding from Kiehl’s.

## Funding support

This research was supported in part by the Intramural Research Program of the National Institutes of Health (NIH), National Eye Institute and the National Center for Advancing Translational Sciences and is subject to the NIH Public Access Policy. Through acceptance of this federal funding, the NIH has been given a right to make the work publicly available in PubMed Central. The contributions of the NIH authors were made as part of their official duties as NIH federal employees, are in compliance with agency policy requirements, and are considered works of the United States government. However, the findings and conclusions presented in this article are those of the authors and do not necessarily reflect the views of the NIH or the US Department of Health and Human Services.

- NIAMS (1 R01 AR077664-01A1) (JHZ).
- Vision for Children Foundation (JHZ).
- National Organization for Albinism and Hypopigmentation Established Researcher Program Grant (JHZ)
- NCI (T32 CA062948) (SJG).

## Supplementary Material

Supplemental data

Supporting data values

## Version 1 06/18/2026

In-Press Preview

## Version 2 08/24/2026

Electronic publication
