# Supplementary material for: Ampyrone is a direct agonist of human tyrosinase and a potential therapeutic for hypopigmentation disorders
Source: JCI Insight. 2026 Jun 18;11(16):e202947. doi: 10.1172/jci.insight.202947 (PMC13502167; doi:10.1172/jci.insight.202947)
Supplement: Supplementary file 1 [file jciinsight-11-202947-s085.pdf]

Ampyrone is a Direct Agonist of Human Tyrosinase and a Potential Therapeutic for Hypopigmentation Disorders.

Supplemental Material

Library	Inhibitors (% of inhibition)		Activators (% of activation)
**Natural products library 1**	Resveratrol (99)<br>4-anilinophenol (99)*<br>Isoliquiritigenin (78)	4-etoxyphenol (50)<br>Hispidin (45)<br>Piceatannol (39)	Phloretin (31)*
**Natural products library 2**	Trichostatin A (86)<br>Polydatin (Piceid) (84)<br>3-9 (35-83)<br>Butein (80)<br>Idronoxil (66)*	Maritimein (66)<br>p-hydroxycinnamaldehyde (62)<br>3',4'-dihydroxyflavone (53)*<br>Marein (36)<br>L-deoxyallin (33)	Sennoside B (31)*<br>Brazilin (42)*
**NCATS Pharmaceutical collection 1**	N-Hydroxybenzamide (111)<br>2-mercaptobenzothiazole (104)<br>Suberoylanilide hydroxamic acid (Vorinostat) (100)<br>5-amino-2-methylophenol (98)<br>4-Hexylresorcinol (92)<br>2,2'-Dithiobisbenzothiazole (88)<br>N-Oxydiethylenebenzothiazole-2-sulfenamide (82)<br>Anethole trithione (78)*<br>Amithiozone (75)<br>Fenaminosulf (72)*<br>1,5-Naphtalenediamine (66)<br>Malachite green oxalate (61)<br>Bisphenol A (BPA) (61)<br>Prothionamide (60)	Thioglycolic acid anilide (57)<br>Ethionamide (50)<br>6-Propyl-2-thiouracil (48)<br>Mafenide hydrochloride (41)<br>4-Chlorobenzoic acid (37)<br>6-Methyl-2-thiouracil (37)<br>Carbaryl (35)<br>Dichloro(ethylenediamine)platinum(II)) (33)<br>Carbocyanine (32)<br>PP242 (31)<br>Thioinosine (31)	4,4'-Thiodianiline (32)
**NCATS Pharmaceutical collection 2**	4-(4-Acetylpiperazin-4-yl)phenol (108)<br>Pantoprazole (105)<br>Omeprazole (104)<br>Lansoprazole (98)<br>Nepaprazole (90)<br>Bufexamac (103)<br>4-Methoxyphenol (70)<br>1,5-Naphthalenediamine (66)<br>Kojic acid (64)<br>Rabeprazole sodium (58)<br>4-(2-Methylbutan-2-yl)phenol (formaldehyde) (99)<br>Allythiourea (52)	Muraglitazar (50)<br>4-tert-Buthylphenol (47)<br>Tenatoprazole (44)<br>Camostat metylate (40)<br>Thiouracil (39)<br>6-Thioguanine (36)*<br>2',4',5'-Trihydroxybutyrophenone (Thbp) (35)<br>Roxindole (34)<br>Nicothiazone (34)<br>Exifone (33)<br>Acetohydroxamic acid (32)<br>Ciclopirox olamine (31)	4-Dimethylaminoantipyrine (Aminopyrine) (31)*<br>Ampyrone (4-Aminoantipyrine) (36)*<br>Hydroxytacrine maleate (105)*
**Genesis collection 1**	1-4 (51-99)		1-2 (30-32)
**Genesis collection 3**	1 (37)		
**Genesis collection 5**	1-15 (31-102)		

Supplemental Table S1. Inhibitors and activators of hTYR^{WT} from a primary HTS. Compounds identified as inhibitors or activators of the hTYR^{WT} from a primary HTS performed with a single test concentration of 115 µM. The compounds indicated by red font were previously described as tyrosinase inhibitors. *Compounds analyzed in this study.

Structure	PC1 Eigenvalue	PC1 Cumulative Proportion of Variance (%)	PC2 Eigenvalue	PC2 Cumulative Proportion of Variance (%)	PC3 Eigenvalue	PC3 Cumulative Proportion of Variance (%)
$hTYR^{WT}$-Unbound	245.0	18.19	168.0	30.67	87.42	37.16
$hTYR^{WT}$-Bound	292.4	21.71	246.4	40.00	97.22	47.21
$hTYR^{P406L}$-Unbound	308.9	22.93	128.8	32.49	80.67	38.48
$hTYR^{P406L}$-Bound	287.2	21.32	178.2	34.55	109.2	42.66

Supplemental Table S2. Principal components summary. This table provides specific values of variance and Eigen used in the modeling of ampyrone bound to $hTYR^{WT}$ and $hTYR^{P406L}$ enzymes.

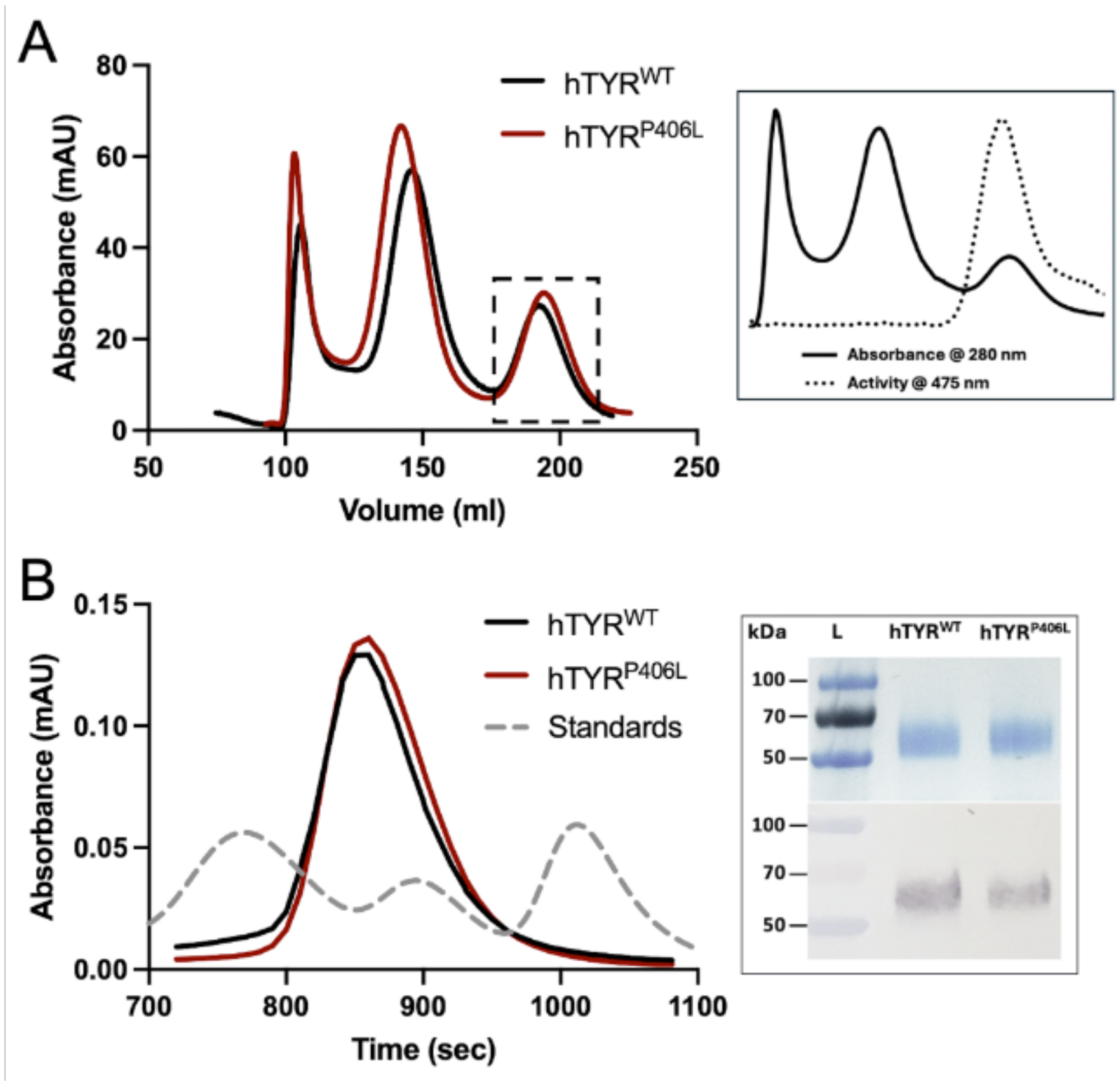

Supplemental Figure S1. Purification of recombinant hTYR^{WT} and hTYR^{P406L} enzymes. A) Size-exclusion chromatography profiles of hTYR^{WT} (black) and hTYR^{P406L} (red) following immobilized metal affinity chromatography (His-Trap Crude 5 mL column) and separation on a HiPrep 26/60 Sephacryl S-300 column. Both hTYR^{WT} and hTYR^{P406L} eluted within the peak indicated by the dotted frame. The inset shows the superimposed absorbance profile recorded at 280 nm (solid line) and the enzymatic activity of the corresponding fractions measured using L-DOPA at 37 ºC and monitored at 475 nm (dotted line), confirms TYR activity exclusively in the last peak. B) Final purification of hTYR^{WT} (black) and hTYR^{P406L} (red) on a Superose 12 GL 10/300 column using a Bio-Logic Duo-Flow Maximizer workstation. The grey dashed line indicates Bio-Rad molecular weight standards (gamma globulin 155 kDa, ovalbumin 44 kDa, and myoglobin 17 kDa). SDS-PAGE (inset, top) demonstrates protein purity and an anti-TYR (T311, 1:2000) Western blotting (inset, bottom) confirms the molecular identity of the purified proteins. Protein ladder bands are indicated at 50, 70, and 100 kDa.

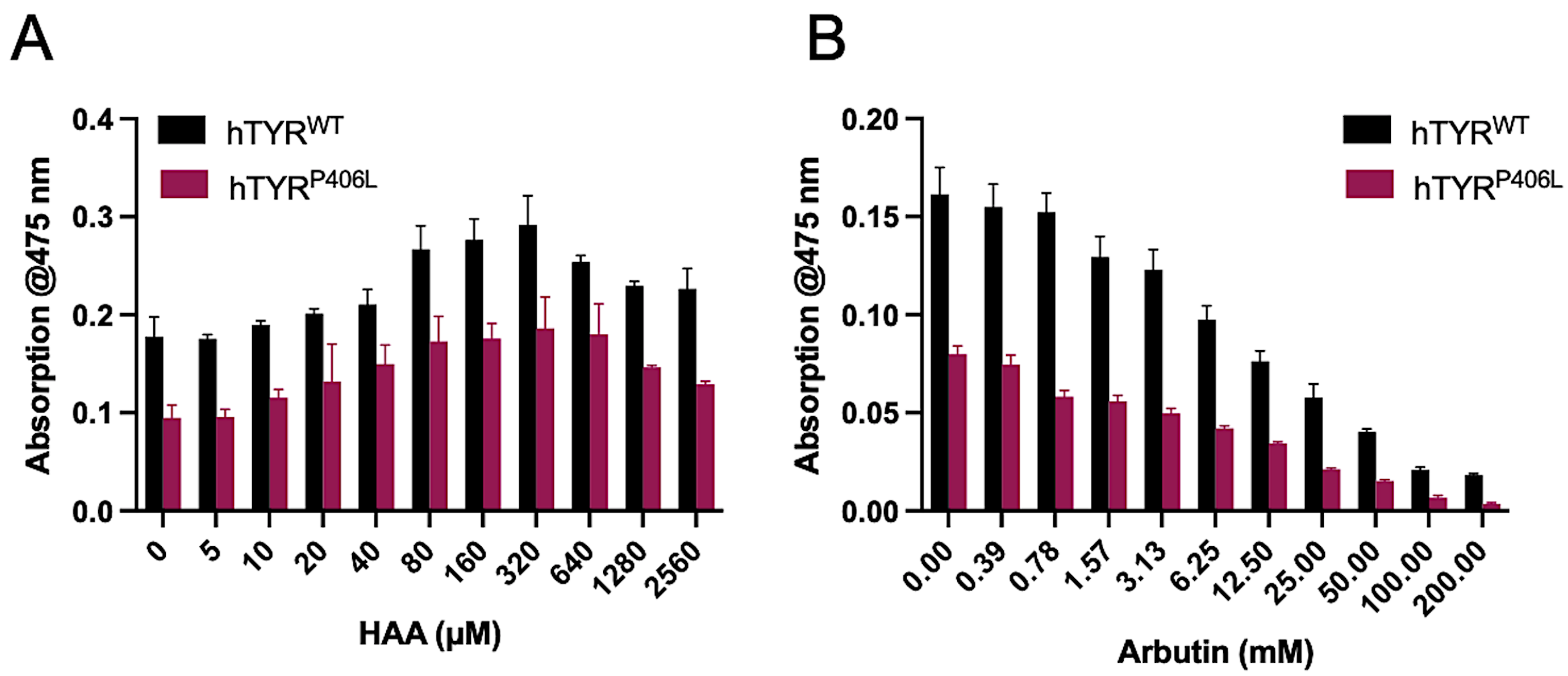

Supplemental Figure S2. Functional validation of hTYR^{WT} and hTYR^{P406L}. Diphenol oxidase activities of hTYR^{WT} (black) and hTYR^{P406L} (red) in the presence of the known activator, 3-hydroxyanthranilic acid (HAA) (A), and the known inhibitor, arbutin (B). Enzymatic activity was measured spectrophotometrically by monitoring absorbance at 475 nm following incubation at 37 °C with 1.5 mM L-DOPA, in the absence or presence of HAA or arbutin at the indicated concentrations.

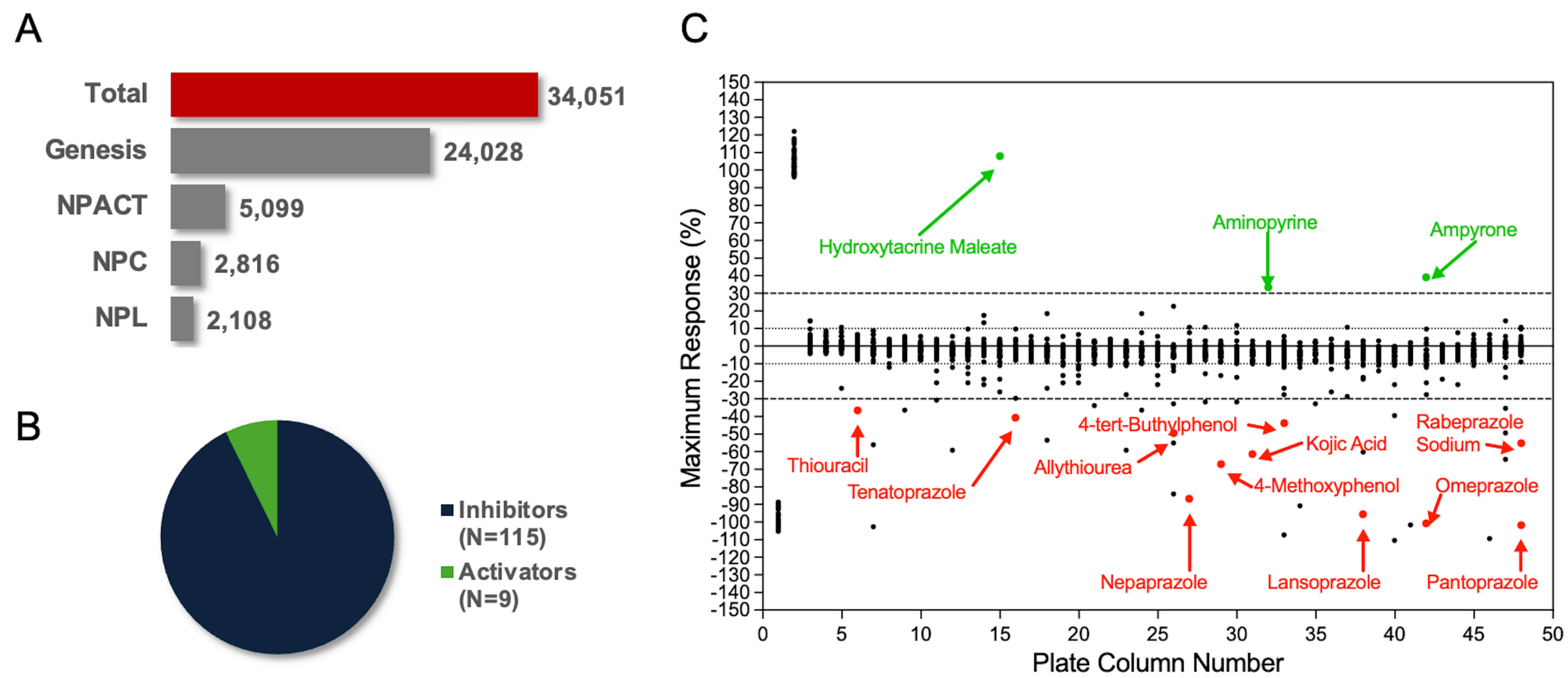

Supplemental Figure S3. Identification of hTYR^{WT} activators and inhibitors from an HTS. A) An HTS of hTYR^{WT} was conducted with a collection of 34,051 compounds from four chemical libraries: the Genesis Diverse Chemical Library (Genesis), the NCATS Pharmacologically Active Chemical Toolbox (NPACT), the NCATS Pharmaceutical Collection (NPC), and the Natural Products Library (NPL) at a single concentration of 115 µM. B) A total of 115 inhibitors and nine activators were identified. C) Activity data resulting from 1,408 compounds of the NCATS Pharmaceutical Collection 2 with names of activators (green) and inhibitors (red) annotated **(see Supplemental Table S1).**

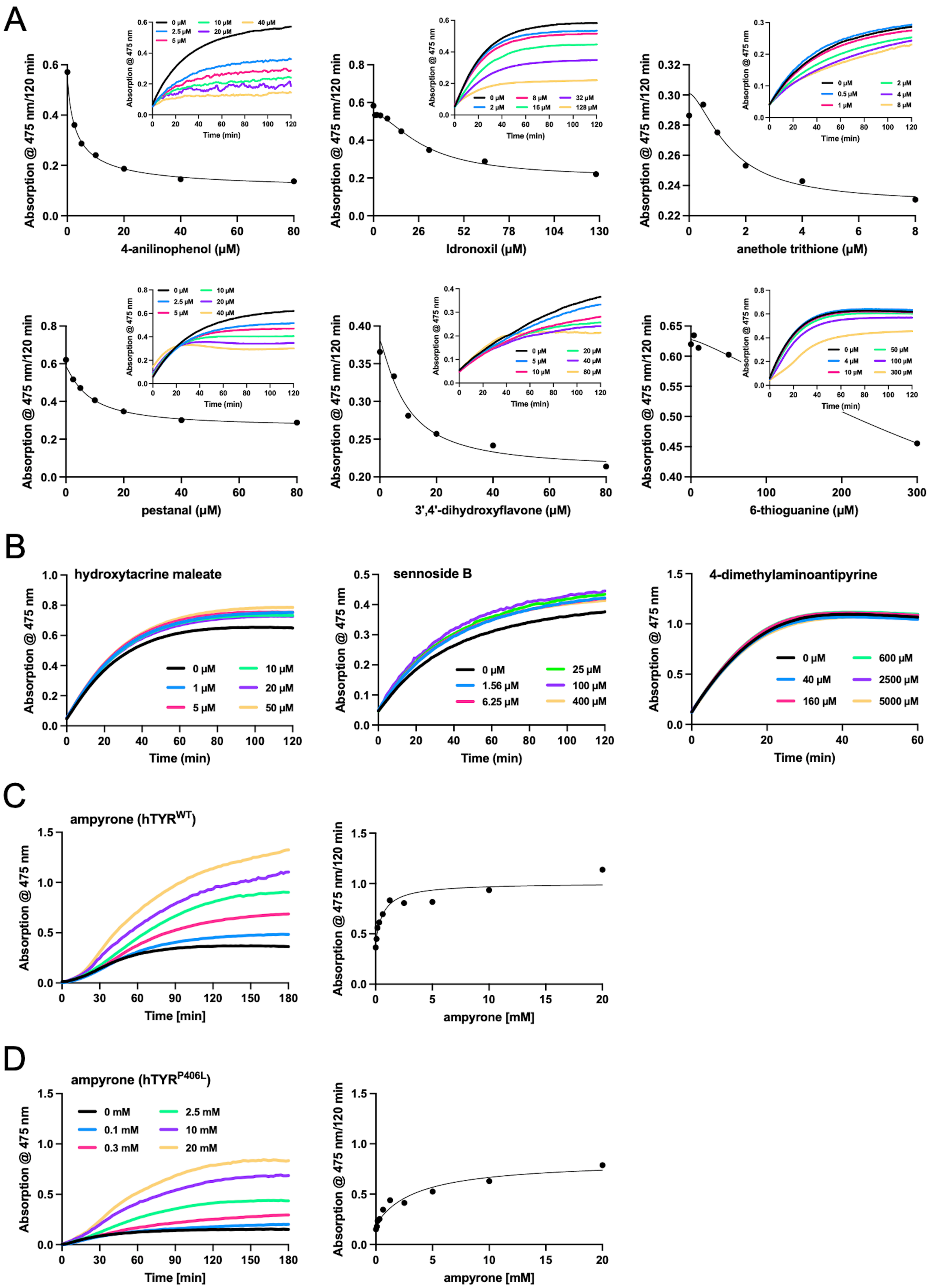
A
Absorption @ 475 nm/120 min
4-anilinophenol (μM)
Idronoxil (μM)
anethole trithione (μM)
pestanal (μM)
3',4'-dihydroxyflavone (μM)
6-thioguanine (μM)
Absorption @ 475 nm
Time (min)
0 μM
2.5 μM
5 μM
10 μM
20 μM
40 μM
2 μM
8 μM
16 μM
32 μM
128 μM
0.5 μM
1 μM
4 μM
80 μM
50 μM
100 μM
300 μM
B
hydroxytacrine maleate
sennoside B
4-dimethylaminoantipyrine
Absorption @ 475 nm
Time (min)
0 μM
1 μM
5 μM
10 μM
20 μM
50 μM
1.56 μM
6.25 μM
25 μM
100 μM
400 μM
40 μM
160 μM
600 μM
2500 μM
5000 μM
C
ampyrone (hTYR^WT)
Absorption @ 475 nm
Time [min]
Absorption @ 475 nm/120 min
ampyrone [mM]
D
ampyrone (hTYR^P406L)
0 mM
0.1 mM
0.3 mM
2.5 mM
10 mM
20 mM
Absorption @ 475 nm
Time [min]
Absorption @ 475 nm/120 min
ampyrone [mM]

Supplemental Figure S4. Confirmation of inhibitor and activator hits. A) Concentration-response graphs for $hTYR^{WT}$ diphenol oxidase activity inhibitors. Absorbance at 475 nm after 120 min was measured following incubation at 37 ºC with increasing concentrations of 4-anilinophenol, idronoxil, anethole trithione, pestanal, 3',4'-dihydroxyflavone, and 6-thioguanine. Data were fitted with a nonlinear regression model to estimate IC_{50}. Insets show time-dependent inhibition profiles for each compound. B) Representative time curves of the $hTYR^{WT}$ diphenol oxidase activity, measured by absorbance at 475 nm, in the presence of the potential activators: hydroxytacrine maleate, sennoside B, and 4-dimethylaminoantipyrine at the indicated concentrations. C) $hTYR^{WT}$ diphenol oxidase activity. Left: representative time curves in the presence of ampyrone. Right: concentration-response curve of ampyrone, absorbance at 475 nm after 120 min following incubation at 37 ºC with increasing concentrations. Data were fitted with a nonlinear regression model to estimate EC_{50}. D) $hTYR^{P406L}$ diphenol oxidase activity. Left: representative time curves in the presence of ampyrone. Right: concentration-response curve of ampyrone, absorbance at 475 nm after 120 min following incubation at 37 ºC with increasing concentrations. Data were fitted with a nonlinear regression model to estimate EC_{50}.

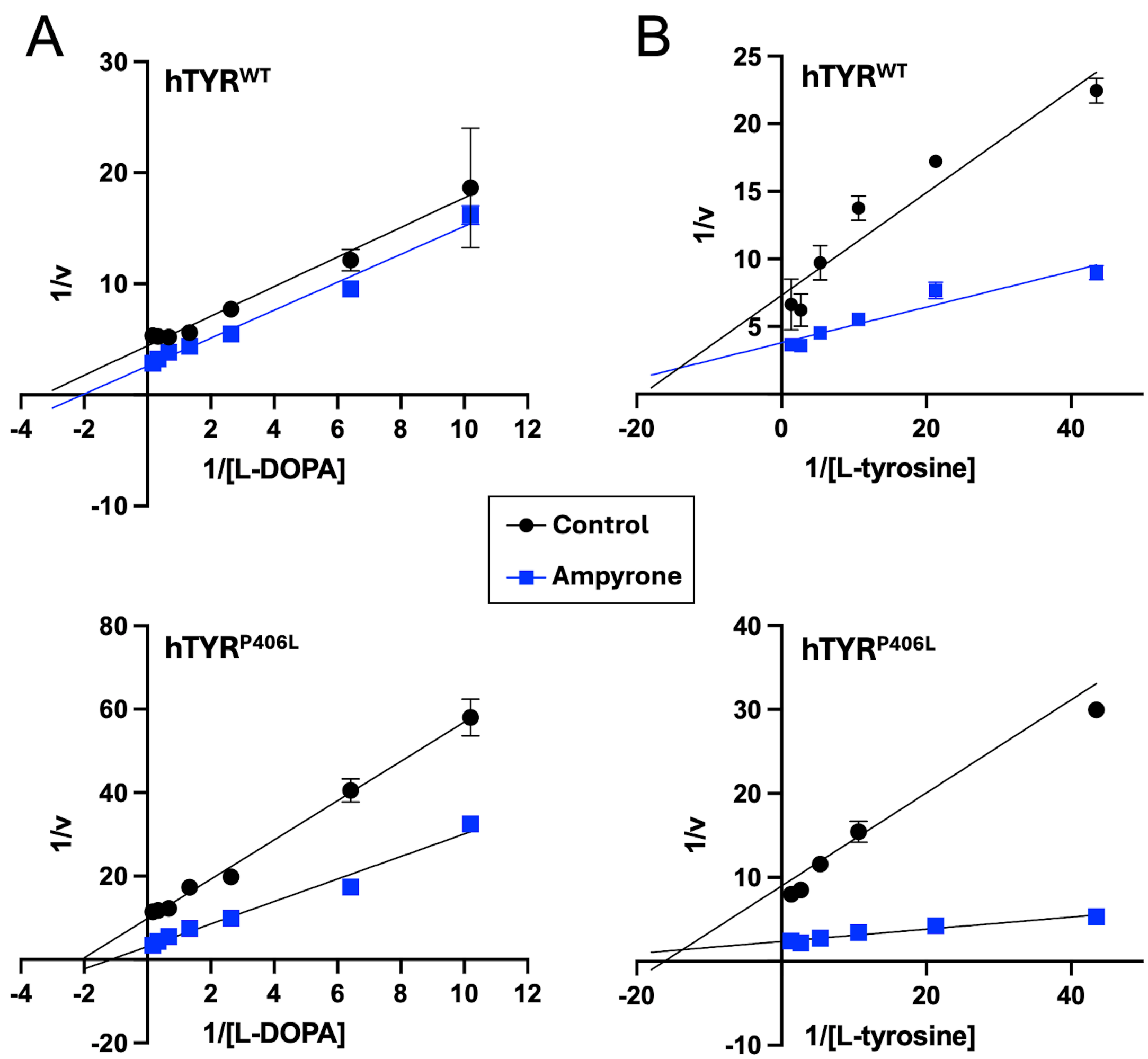

Supplemental Figure S5. Lineweaver-Burk analysis of ampyrone's effect on the diphenol oxidase and monophenolase activity of hTYR^{WT} and hTYR^{P406L}. Lineweaver-Burk plots of diphenol oxidase (A) and monophenolase (B) activity of hTYR^{WT} (top panels) and hTYR^{P406L} (bottom panels) enzymes measured at 37 °C in the presence (ampyrone, blue) or absence (control, black) of 10 mM ampyrone, using increasing concentrations of L-DOPA or L-tyrosine, respectively. Data points were fit to the linearized Lineweaver-Burk equation in GraphPad Prism 10. Data represent the mean ± SD from three replicate experiments.

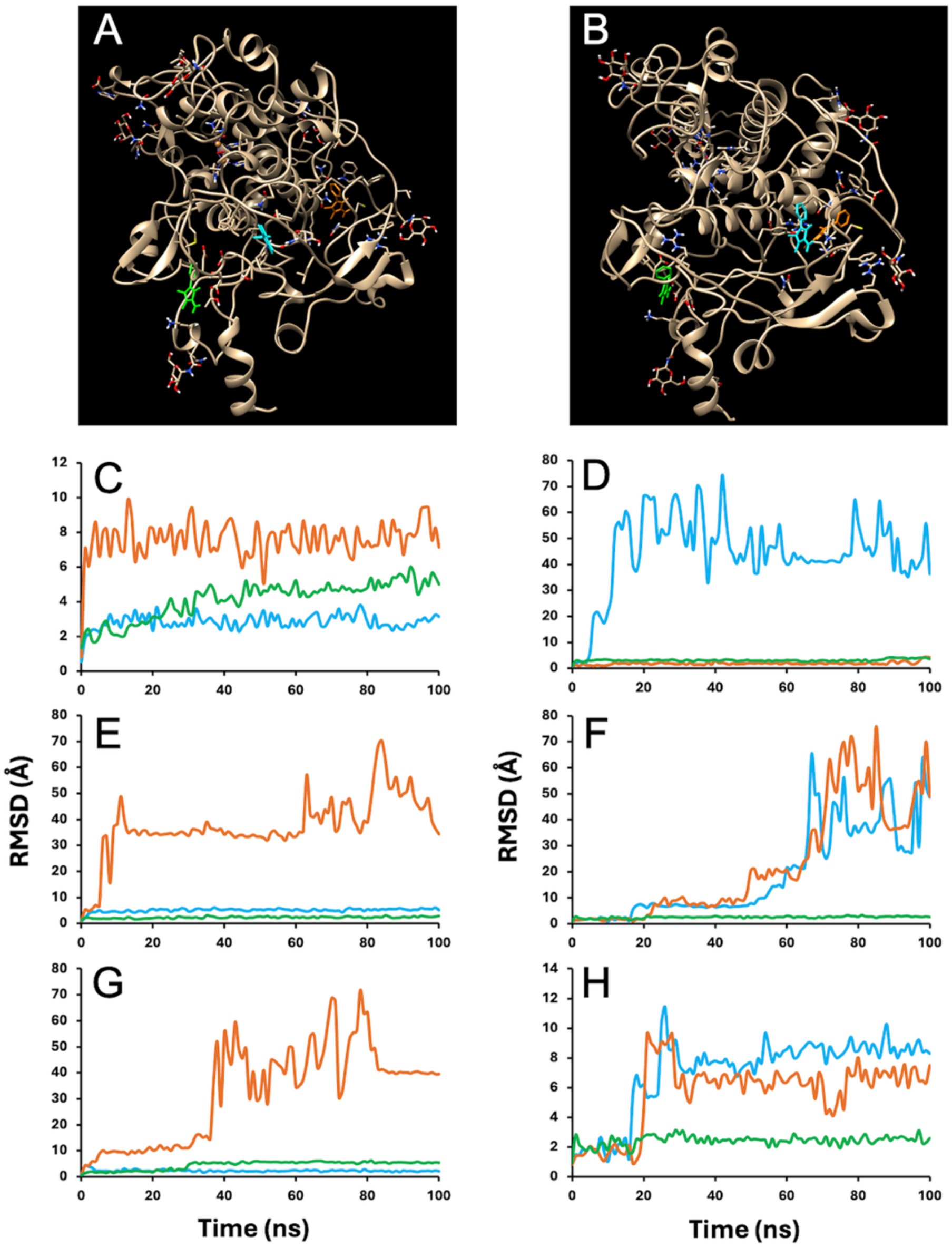

Supplemental Figure S6. Ampyrone binding sites and stability across molecular dynamics simulations. The initial locations of ampyrone binding sites in $hTYR^{WT}$ (A) and $hTYR^{P406}$ (B) are shown. Three ampyrone molecules are depicted in blue (A1), orange (A2), and green (A3). RMSD profiles are shown in panels C-H, with trajectory colors corresponding to those used in panels A and B. RMSD profiles for ampyrone molecules bound to $hTYR^{WT}$ across three independent simulation replicas are shown in panels C, D, and E. RMSD profiles for ampyrone molecules bound to the $hTYR^{P406L}$ variant across three independent simulation replicas are shown in panels F, G, and H.

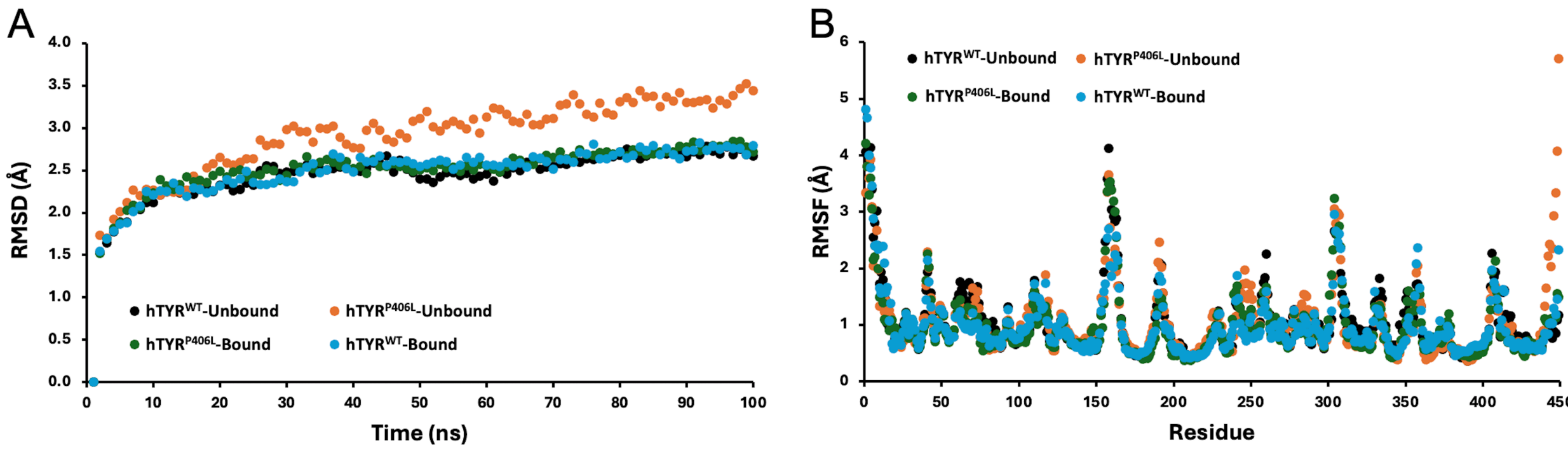

Supplemental Figure S7. Comparison of time-dependent changes in the presence and absence of ampyrone binding for hTYR^{WT} and hTYR^{P406L}. RMSD and RMSF profiles are shown in panels A and B, respectively. RMSD and RMSF trajectories for the unbound proteins are shown in black and orange, whereas those for the bound state are shown in green and blue.

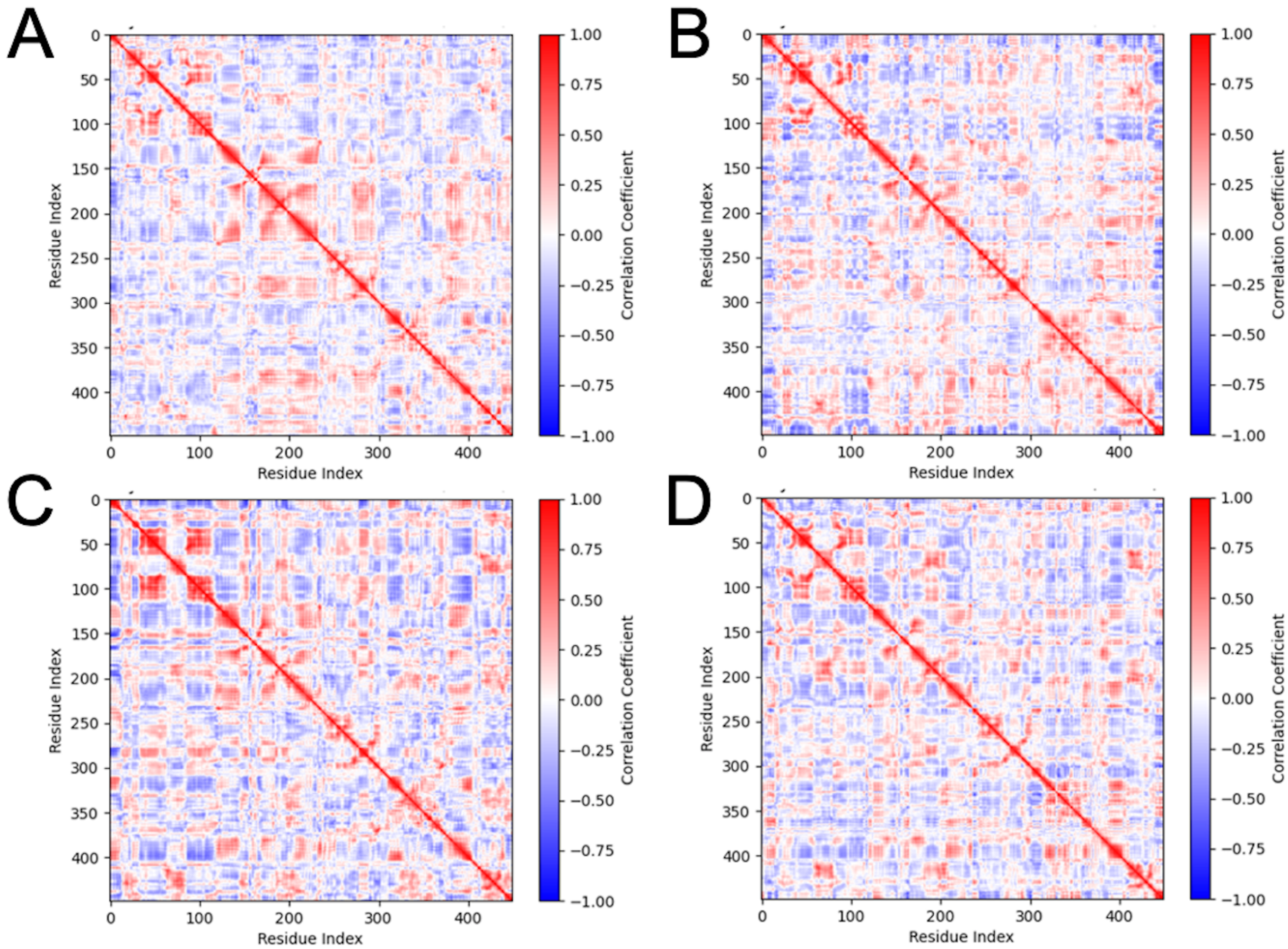

Supplemental Figure S8. Dynamical cross-correlation matrices (DCCM) for hTYR^{WT} and hTYR^{P406L}. DCCM for hTYR^{WT} without ampyrone (A), hTYR^{P406L} without ampyrone (B), hTYR^{WT} with ampyrone (C), and hTYR^{P406L} with ampyrone (D).

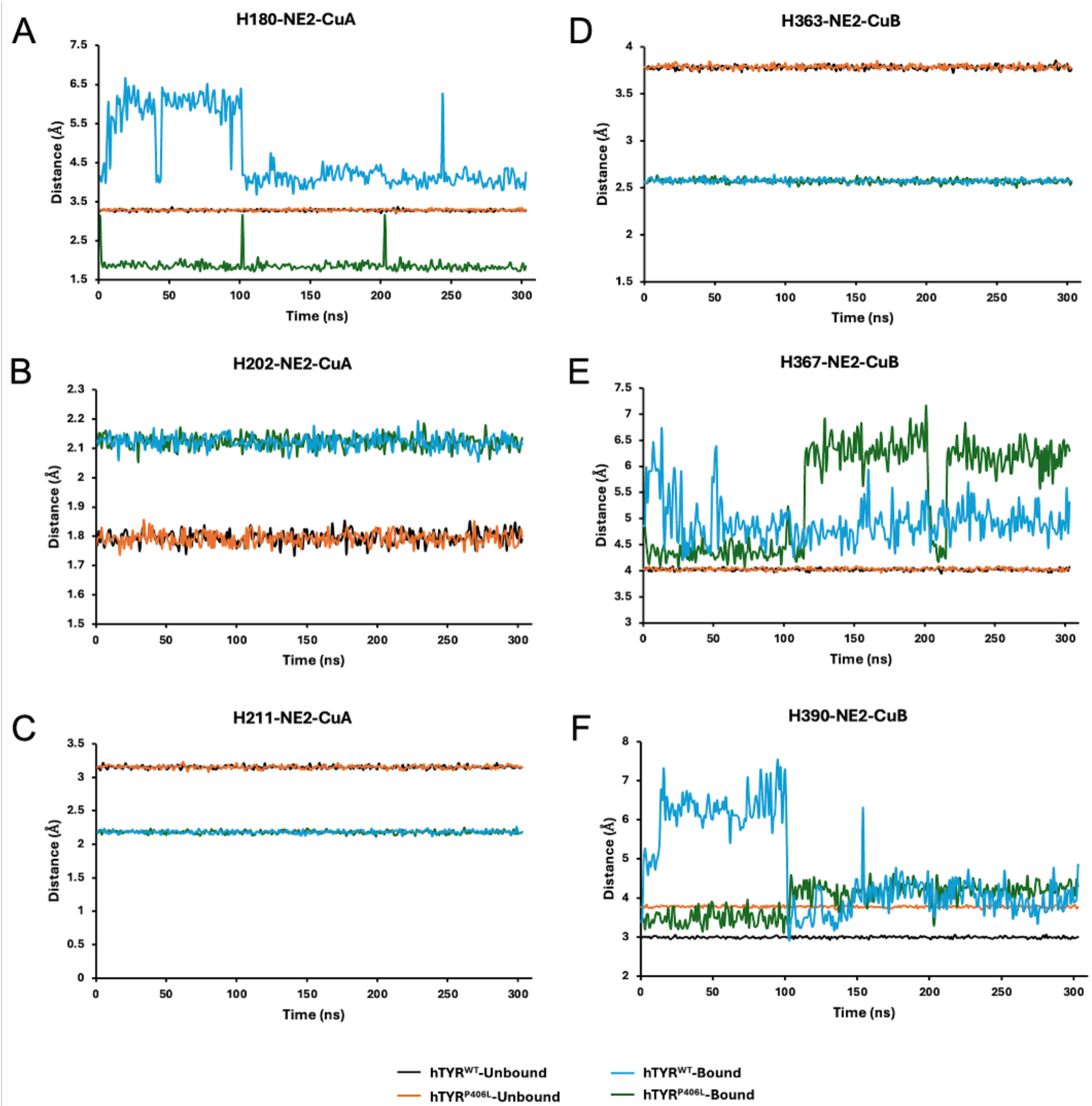

Supplemental Figure S9. Distances between six histidine residues coordinating active site coppers (CuA and CuB). For each protein two states are considered, 'Bound' (ampyrone is bound to hTYR) and 'Unbound' (no ampyrone binding). The graphs show the distances between the CuA and the NE2 atom of H180 (A), H202 (B), and H211 (C), as well as the CuB and the NE2 atom of H363 (D), H367 (E), and H390 (F) for hTYR^{WT}-Unbound (black), hTYR^{P406L}-Unbound (orange), hTYR^{WT}-Bound (blue), hTYR^{P406L}-Bound (green).

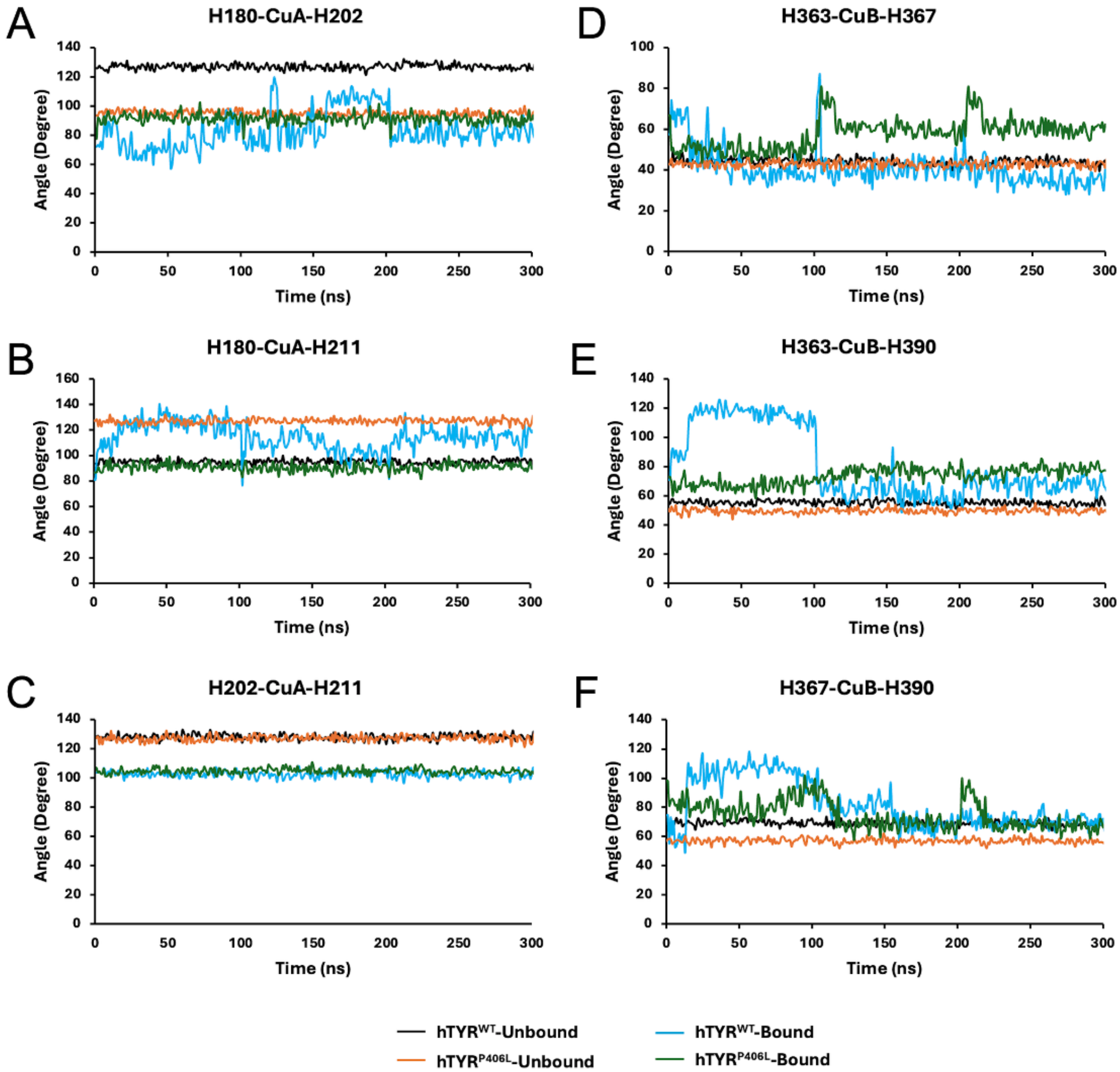

Supplemental Figure S10. Angles of copper coordination for six histidine residues in the TYR active site. The graphs show the coordination angles between the CuA and H180/H202 (A), H180/H211 (B), H202/H211 (C), and between CuB and H363/H367 (D), H363/H390 (E), and H367/H390 (F) for hTYR^{WT}-Unbound (black), hTYR^{P406L}-Unbound (orange), hTYR^{WT}-Bound (blue), hTYR^{P406L}-Bound (green).

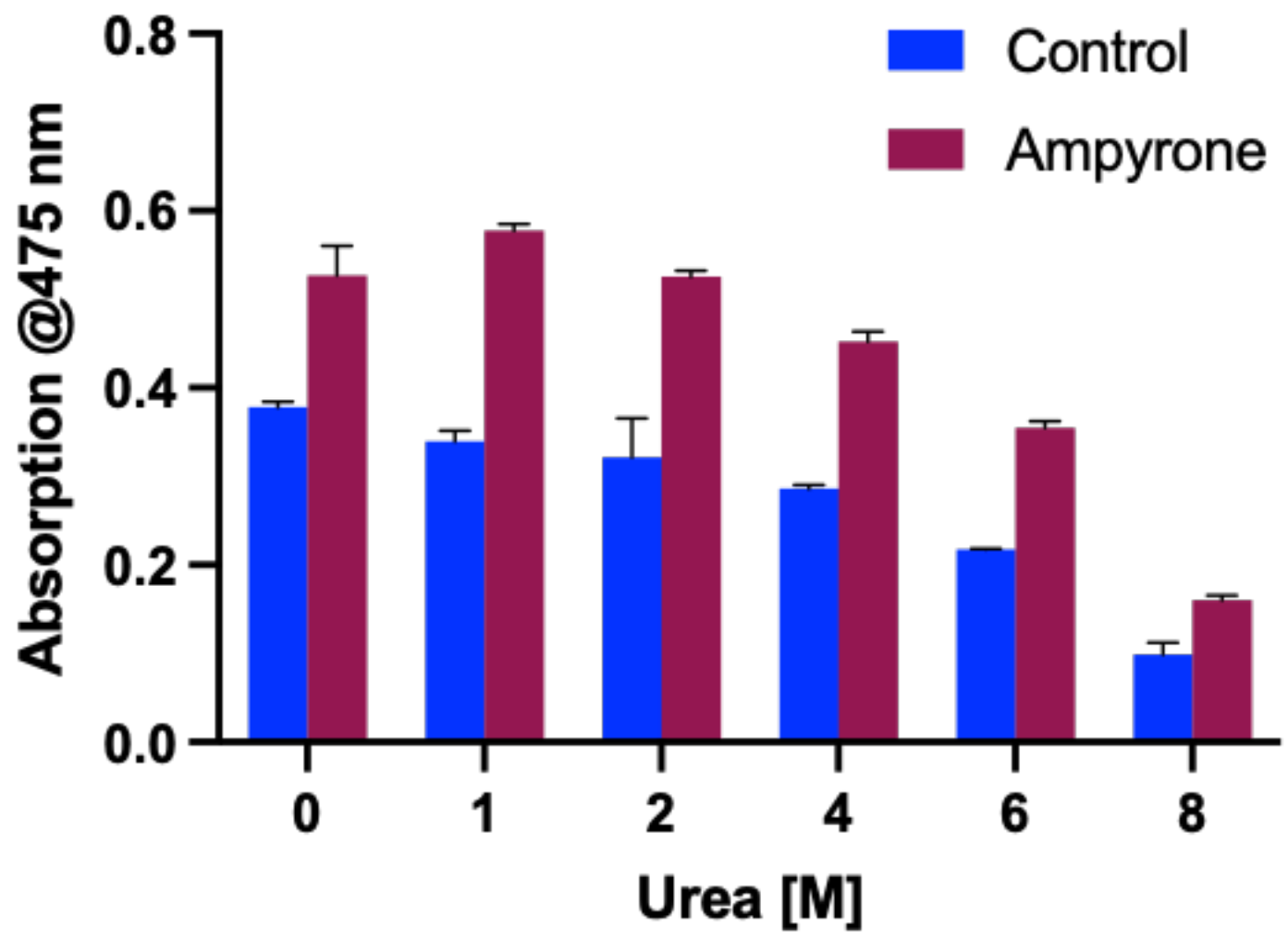

Supplemental Figure S11. Protective effect of ampyrone on hTYR stability. Diphenol oxidase activity of $hTYR^{WT}$ (control, blue bars) and with 5 mM ampyrone (red bars) measured as absorbance at 475 nm after a 1-hour incubation with urea at concentrations ranging from 0 to 8 M. Error bars represent standard deviations from three replicate experiments. Absorbance of the blank (samples without enzyme) was subtracted from all values.

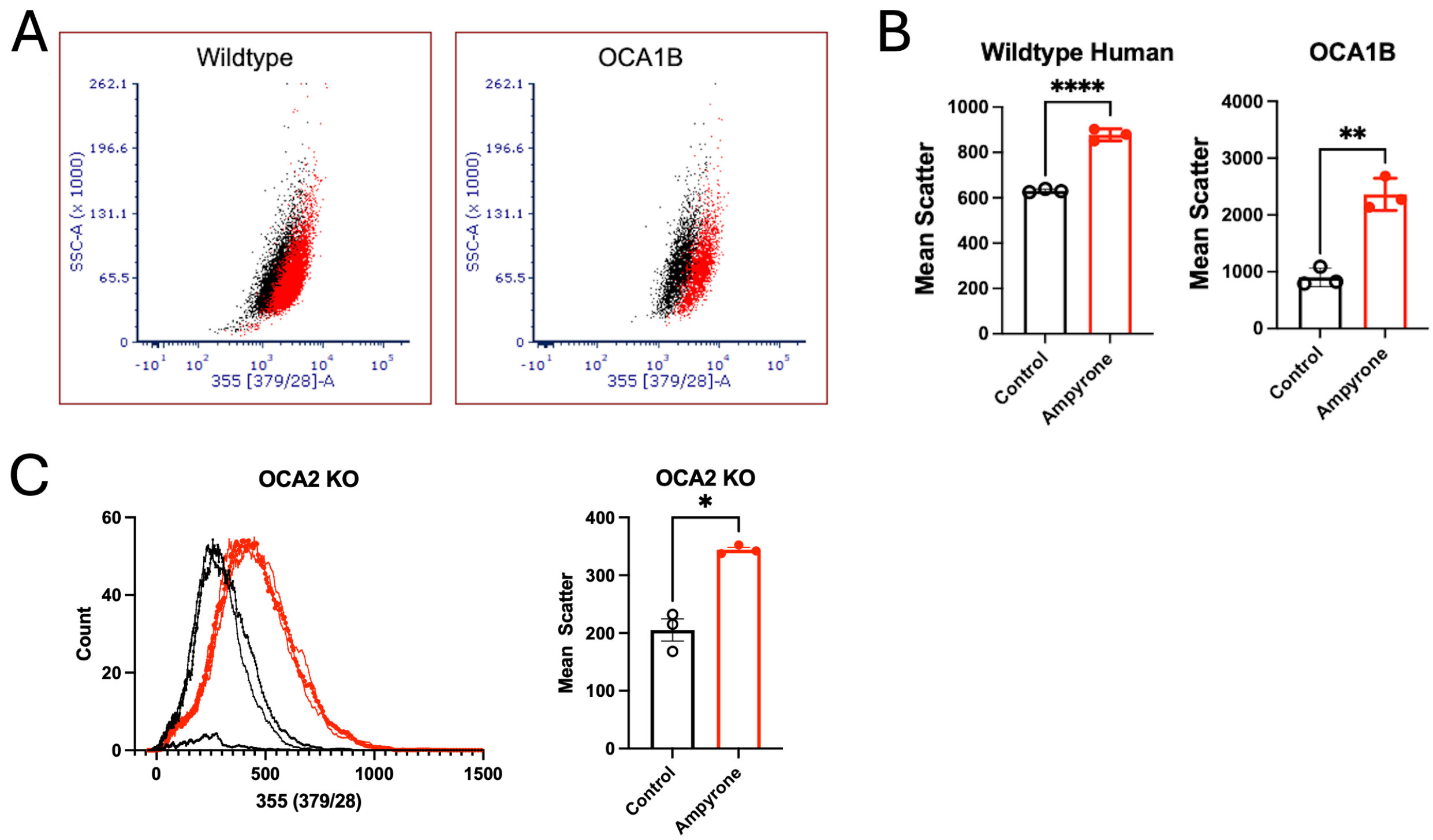

Supplemental Figure S12. Ampyrone induces melanin synthesis in melanocytes. A) Scatter plot of human wild-type (C4) and OCA1B-1125 melanocytes (see Figure 5 for histogram and mean scatter for these data) after 48 hours of treatment with control (black) and ampyrone (red) showing side scatter on the Y axis and scatter at 355 nm on X axis. B) Mean scatter at 355 nm for an additional set of wild-type human (NBMEL 1284) and OCA1B (OCA1B-1235) human melanocytes after treatment with vehicle (control, black) and ampyrone (0.2 mM, red) for 48 hours. Scatter plots for panel B are not shown. C) Counts of OCA2 null mouse melanocytes with scatter at 355 nm (left panel) and mean scatter intensity (right panel) treated with vehicle (black) or ampyrone (0.2 mM, red) for 48 hours. Scatter plots for panel C are not shown. Each point on the mean scatter plots is a biological replicate. Representative experiment performed three times. Student's t-test, unpaired $N \geq 3$ (*$P<0.05$, **$P<0.01$ ****$P<0.0001$).

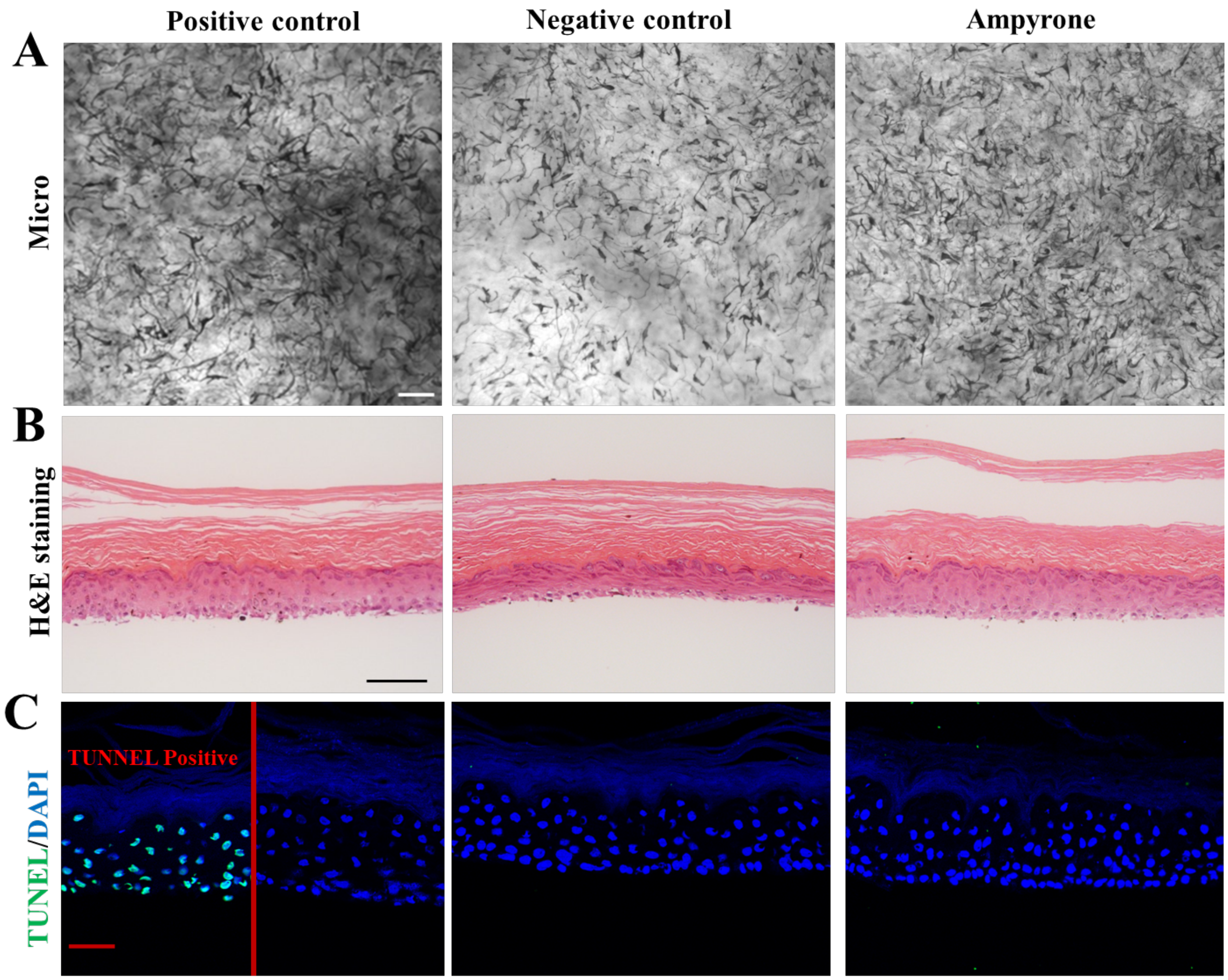

Supplemental Figure S13. Ampyrone treatment effect on the morphology of 3D epidermal cultures. A) Inverted microscopic images showing representative melanocyte pigmentation and cellular morphology in positive control (αMSH/βFGF), ampyrone treated and negative control wells. B) Hematoxylin and eosin (H&E) staining showing the preserved epidermal structure across three groups after treatment. C) TUNEL staining (green) of 3D epidermal sections counterstained with DAPI (blue), demonstrating no apoptotic nuclei in any experimental samples. The validity of the TUNEL assay was confirmed by pre-treatment of sections with DNAse I (left subpanel in C). Scale bars: A and B, 100 μm; C, 50 μm.
